# Supplementary material for: Repetitive in vivo manual loading of the spine elicits cellular responses in porcine annuli fibrosi
Source: PLoS One. 2021 Mar 23;16(3):e0248104. doi: 10.1371/journal.pone.0248104 (PMC7987143; doi:10.1371/journal.pone.0248104)
Supplement: S1 Table — (PDF) [file pone.0248104.s001.pdf]

Ensembl gene ID	Gene name	Gene description	C	Tx	FC	P
ENSSSCG00000004928	CILP	cartilage intermediate layer protein	1019	69.5	14.3	0.01
ENSSSCG00000012848	Eps8l2	EPS8 like 2	1.1	0.1	14.3	0.01
ENSSSCG00000028015	GAL3ST3	galactose-3-O-sulfotransferase 3	0.8	0.1	14.3	< 0.01
ENSSSCG00000004225	TPD52L1	tumor protein D52-like 1	24.8	2.0	12.5	0.01
ENSSSCG00000028776			12.6	1.1	11.1	< 0.01
ENSSSCG00000000749	SLC6A12	solute carrier family 6 member 12	9.7	0.9	11.1	< 0.01
ENSSSCG00000027515	FCGBP	Fc fragment of IgG binding protein	0.3	0.0	11.1	< 0.01
ENSSSCG00000000716	KCNA1	potassium voltage-gated channel subfamily A member 1	13.5	1.4	10.0	< 0.01
ENSSSCG00000025667	FBXO2	F-box protein 2	5.8	0.6	10.0	< 0.01
ENSSSCG00000008263	M1AP	meiosis 1 associated protein	3.2	0.4	9.1	0.01
ENSSSCG00000031053	S100A1	S100 calcium binding protein A1	1865	224.2	8.3	< 0.01
ENSSSCG00000025523		collagen type II alpha 1 chain	100663	13829	7.1	0.01
ENSSSCG00000011413	C13H3orf18	chromosome 3 open reading frame 18	7.0	1.0	7.1	< 0.01
ENSSSCG00000026142	TNK1	tyrosine kinase non receptor 1	0.8	0.1	7.1	0.01
ENSSSCG00000011700	CP	ceruloplasmin	574.7	86.3	6.7	< 0.01
ENSSSCG00000016295	NGEF	neuronal guanine nucleotide exchange factor	78.6	11.8	6.7	< 0.01
ENSSSCG00000029868			0.9	0.1	6.7	0.01
ENSSSCG00000004191	MOXD1	monooxygenase DBH like 1	35.2	5.7	6.3	< 0.01
ENSSSCG00000004422	WISP3	WNT1 inducible signaling pathway protein 3	18.0	2.9	6.3	< 0.01
ENSSSCG00000002764	SMPD3	sphingomyelin phosphodiesterase 3	12.9	2.0	6.3	< 0.01
ENSSSCG00000022609		WW domain containing E3 ubiquitin protein ligase 2	523.9	90.5	5.9	< 0.01
ENSSSCG00000013869	CPAMD8	C3 and PZP like, alpha-2-macroglobulin domain containing 8	146.3	24.5	5.9	< 0.01
ENSSSCG00000028395			7.5	1.3	5.9	< 0.01
ENSSSCG00000027992	MST1R	macrophage stimulating 1 receptor	3.6	0.6	5.9	0.01
ENSSSCG00000004017	FRMD1	FERM domain containing 1	1.5	0.3	5.9	0.01
ENSSSCG00000027710	PDZD3	PDZ domain containing 3	0.4	0.1	5.9	< 0.01
ENSSSCG00000001473	COL11A2	collagen type XI alpha 2 chain	2822	515.7	5.6	0.01
ENSSSCG00000013403	GALNT18	polypeptide N-acetylgalactosaminyltransferase 18	156.3	28.8	5.6	0.01
ENSSSCG00000028203	ICA	porcine inhibitor of carbonic anhydrase	30.2	5.3	5.6	< 0.01
ENSSSCG00000016513		KIAA1549	8.3	1.5	5.6	0.01

Ensembl gene ID	Gene name	Gene description	C	Tx	FC	P
ENSSSCG00000014908	CCDC89	coiled-coil domain containing 89	3.2	0.6	5.6	< 0.01
ENSSSCG00000023749	ADM2	adrenomedullin 2	2.1	0.4	5.6	< 0.01
ENSSSCG00000005002	MDGA2		1.3	0.2	5.6	< 0.01
ENSSSCG00000023329	APC2	adenomatosis polyposis coli 2	0.8	0.2	5.6	< 0.01
ENSSSCG00000001832	ACAN	aggrecan	2029	386.0	5.3	0.01
ENSSSCG00000027787			60.6	11.3	5.3	< 0.01
ENSSSCG00000012975	SNX32	sorting nexin 32	3.8	0.7	5.3	< 0.01
ENSSSCG00000015223	LOC100523329	ATP-dependent RNA helicase DDX25	3.0	0.6	5.3	< 0.01
ENSSSCG00000011216	LRRC3B	leucine rich repeat containing 3B	2.8	0.5	5.3	0.01
ENSSSCG00000016780	LOC100517978		2.0	0.4	5.3	< 0.01
ENSSSCG00000010371	ANTXRL	anthrax toxin receptor-like	1.4	0.3	5.3	< 0.01
ENSSSCG00000002028	CMTM5	CKLF-like MARVEL transmembrane domain containing 5	3.5	0.7	5.0	0.01
ENSSSCG00000029011	LOC100622932	potassium voltage-gated channel subfamily A member 4	1.6	0.3	5.0	< 0.01
ENSSSCG00000025021	MALL	T-cell differentiation protein-like	64.2	13.4	4.8	0.01
ENSSSCG00000028237	POU2F3	POU class 2 homeobox 3	1.1	0.2	4.8	0.01
ENSSSCG00000004584	GCNT3	glucosaminyl (N-acetyl) transferase 3, mucin type	1.0	0.2	4.8	< 0.01
ENSSSCG00000029582	SCRG1	stimulator of chondrogenesis 1	1777	391.1	4.5	< 0.01
ENSSSCG00000010370	ANXA8		1056	234.3	4.5	< 0.01
ENSSSCG00000026551			573.0	124.0	4.5	< 0.01
ENSSSCG00000005315	CA9	carbonic anhydrase 9	62.8	13.7	4.5	< 0.01
ENSSSCG00000023229		ETS variant 5	39.9	8.6	4.5	< 0.01
ENSSSCG00000021860		histone cluster 1 H4 family member b	5.8	1.3	4.5	< 0.01
ENSSSCG00000009388	KCNRG	potassium channel regulator	1.4	0.3	4.5	< 0.01
ENSSSCG00000026378	LOC100738939	nuclear envelope integral membrane protein 1	0.8	0.2	4.5	< 0.01
ENSSSCG00000007387	LOC396757		3.1	0.7	4.3	0.01
ENSSSCG00000027827	GALE	UDP-galactose-4-epimerase	76.9	18.4	4.2	< 0.01
ENSSSCG00000008062			11.6	2.8	4.2	< 0.01
ENSSSCG00000011098	LOC100512980		2.8	0.7	4.2	0.01
ENSSSCG00000010589	SFXN2	sideroflexin 2	2.5	0.6	4.2	< 0.01
ENSSSCG00000014634	C9H11orf42	chromosome 11 open reading frame 42	1.7	0.4	4.2	< 0.01

Ensembl gene ID	Gene name	Gene description	C	Tx	FC	P
ENSSSCG00000025522			1.1	0.3	4.2	0.01
ENSSSCG00000030827	FGFR3	fibroblast growth factor receptor 3	47.1	11.9	4.0	< 0.01
ENSSSCG00000003472	ARHGEF19	Rho guanine nucleotide exchange factor 19	38.4	9.8	4.0	< 0.01
ENSSSCG00000022590	LOC106505387	G protein-coupled receptor class C group 5 member C	20.5	5.2	4.0	0.01
ENSSSCG00000027929			13.0	3.3	4.0	< 0.01
ENSSSCG00000002847	GPT2	glutamic--pyruvic transaminase 2	9.9	2.4	4.0	0.01
ENSSSCG00000014395	PCDH12	protocadherin 12	7.0	1.7	4.0	< 0.01
ENSSSCG00000021852	FAM124B	family with sequence similarity 124 member B	2.5	0.6	4.0	0.01
ENSSSCG00000003214	KCNC3	potassium voltage-gated channel subfamily C member 3	0.3	0.1	4.0	0.01
ENSSSCG00000016034	COL3A1	collagen, type III, alpha 1	11556	3006	3.8	< 0.01
ENSSSCG00000008723	HTRA3	HtrA serine peptidase 3	236.1	60.4	3.8	< 0.01
ENSSSCG00000022685	ROM1	retinal outer segment membrane protein 1	52.3	13.4	3.8	< 0.01
ENSSSCG00000013335	LGR4	leucine rich repeat containing G protein-coupled receptor 4	23.9	6.3	3.8	< 0.01
ENSSSCG00000014439			15.6	4.1	3.8	< 0.01
ENSSSCG00000002637	DBNDD1		2.1	0.6	3.8	< 0.01
ENSSSCG00000003440	AADACL4	arylacetamide deacetylase like 4	1.9	0.5	3.8	< 0.01
ENSSSCG00000001131	BTN2A2	butyrophilin subfamily 2 member A2	1.9	0.5	3.8	0.01
ENSSSCG00000006957	RHPN1	rhophilin Rho GTPase binding protein 1	1.8	0.5	3.8	0.01
ENSSSCG00000000121	LOC100623444	galanin receptor 3	1.1	0.3	3.8	< 0.01
ENSSSCG00000026242			0.9	0.2	3.8	0.01
ENSSSCG00000025826	BOC	BOC cell adhesion associated, oncogene regulated	69.6	18.7	3.7	0.01
ENSSSCG00000026427		RAR related orphan receptor C	7.5	2.0	3.7	< 0.01
ENSSSCG00000005710		laminin subunit gamma 3	6.6	1.8	3.7	< 0.01
ENSSSCG00000017537	HOXB8	homeobox B8	6.2	1.7	3.7	< 0.01
ENSSSCG00000000699			4.1	1.1	3.7	< 0.01
ENSSSCG00000017188	ZACN	zinc activated ion channel	1.5	0.4	3.7	< 0.01
ENSSSCG00000030906	MPL	MPL proto-oncogene, thrombopoietin receptor	1.1	0.3	3.7	< 0.01
ENSSSCG00000007807	CD19	CD19 molecule	0.7	0.2	3.7	< 0.01
ENSSSCG00000030217	COLGALT2	collagen beta (1-O)galactosyltransferase 2	69.0	19.5	3.6	< 0.01
ENSSSCG00000011922	LOC100739776		59.3	16.8	3.6	0.01

Ensembl gene ID	Gene name	Gene description	C	Tx	FC	P
ENSSSCG00000024158	LOC100738137	anoctamin-1	15.8	4.5	3.6	< 0.01
ENSSSCG00000005320	RGP1	RGP1 homolog, RAB6A GEF complex partner 1	10.2	2.9	3.6	0.01
ENSSSCG00000013934	CILP2	cartilage intermediate layer protein 2	2259	648.0	3.4	< 0.01
ENSSSCG00000002997	MIA		329.6	94.5	3.4	< 0.01
ENSSSCG00000012482	SRPX2	sushi repeat containing protein, X-linked 2	239.4	69.7	3.4	< 0.01
ENSSSCG00000015551	RGS8	regulator of G-protein signaling 8	8.1	2.3	3.4	0.01
ENSSSCG00000012950	RIN1	Ras and Rab interactor 1	2.3	0.7	3.4	< 0.01
ENSSSCG00000028672	GCKR	glucokinase regulator	1.3	0.4	3.4	< 0.01
ENSSSCG00000003341	TAS1R3	taste 1 receptor member 3	1.3	0.4	3.4	< 0.01
ENSSSCG00000030676	KRTCAP3	keratinocyte associated protein 3	1.1	0.3	3.4	< 0.01
ENSSSCG00000001634			0.9	0.3	3.4	< 0.01
ENSSSCG00000017569	CHAD	chondroadherin	2116	643.9	3.3	0.01
ENSSSCG00000026943	MRAP2	melanocortin 2 receptor accessory protein 2	47.0	14.2	3.3	< 0.01
ENSSSCG00000016900	ESM1		26.9	8.1	3.3	< 0.01
ENSSSCG00000022472			16.4	4.9	3.3	0.01
ENSSSCG00000003802	SLC35D1		14.4	4.4	3.3	< 0.01
ENSSSCG00000029180	KCTD7	potassium channel tetramerization domain containing 7	11.2	3.4	3.3	< 0.01
ENSSSCG00000024470	LOC100621284	uncharacterized LOC100621284	9.9	2.9	3.3	< 0.01
ENSSSCG00000006639	C4H1orf56	chromosome 1 open reading frame 56	1.2	0.4	3.3	< 0.01
ENSSSCG00000014845			1.1	0.3	3.3	0.01
ENSSSCG00000029541		pleckstrin and Sec7 domain containing 2	0.8	0.3	3.3	< 0.01
ENSSSCG00000008038	SLC9A3R2	Na (+) exchange regulatory cofactor	52.9	16.5	3.2	< 0.01
ENSSSCG00000002368	LTBP2	latent transforming growth factor beta binding protein 2	20.3	6.2	3.2	0.01
ENSSSCG00000008036	SYNGR3	synaptogyrin 3	14.7	4.5	3.2	0.01
ENSSSCG00000012869	LOC100518411		10.7	3.3	3.2	< 0.01
ENSSSCG00000028240			9.5	2.9	3.2	0.01
ENSSSCG00000002546	XRCC3	X-ray repair cross complementing 3	4.7	1.4	3.2	< 0.01
ENSSSCG00000015015	ARHGAP20	Rho GTPase activating protein 20	1.7	0.5	3.2	< 0.01
ENSSSCG00000027764	LOC102166519	uncharacterized LOC102166519	35.0	11.2	3.1	< 0.01
ENSSSCG00000020945	SMDT1	single-pass membrane protein with aspartate rich tail 1	29.1	9.4	3.1	< 0.01

Ensembl gene ID	Gene name	Gene description	C	Tx	FC	P
ENSSSCG00000015398	LOC100739844		22.7	7.3	3.1	< 0.01
ENSSSCG00000017379	ETV4	ETS variant 4	23.0	7.3	3.1	< 0.01
ENSSSCG00000020906	TNFSF10	tumor necrosis factor superfamily member 10	17.3	5.6	3.1	0.01
ENSSSCG00000008021	TMEM204	transmembrane protein 204	12.0	3.8	3.1	< 0.01
ENSSSCG00000000807	SLC38A1	solute carrier family 38 member 1	5.4	1.7	3.1	0.01
ENSSSCG00000021874	LOC102163523		4.3	1.4	3.1	0.01
ENSSSCG00000029257	LOC396905	acrosin inhibitor	2.7	0.9	3.1	< 0.01
ENSSSCG00000010086		coiled-coil domain containing 116	1.3	0.4	3.1	0.01
ENSSSCG00000017478	RAPGEFL1	Rap guanine nucleotide exchange factor like 1	1.0	0.3	3.1	< 0.01
ENSSSCG00000003108	NPAS1	neuronal PAS domain protein 1	0.6	0.2	3.1	< 0.01
ENSSSCG00000015083	FXYD6	FXYD domain containing ion transport regulator 6	195.8	65.2	3.0	0.01
ENSSSCG00000004485	CD109	CD109 molecule	141.8	47.2	3.0	< 0.01
ENSSSCG00000008266	LOXL3		115.6	38.1	3.0	0.01
ENSSSCG00000028092	GNG2	G protein subunit gamma 2	47.6	15.6	3.0	< 0.01
ENSSSCG00000021997	ALS2CL		29.1	9.6	3.0	< 0.01
ENSSSCG00000024888			18.9	6.3	3.0	0.01
ENSSSCG00000005657	PKN3	protein kinase N3	13.5	4.5	3.0	< 0.01
ENSSSCG00000005490			8.5	2.8	3.0	< 0.01
ENSSSCG00000005361	ALDH1B1	aldehyde dehydrogenase 1 family member B1	3.9	1.3	3.0	0.01
ENSSSCG00000025892	LOC100513290	proline rich 3	2.7	0.9	3.0	0.01
ENSSSCG00000007232	DUSP15		1.4	0.5	3.0	0.01
ENSSSCG00000029940		potassium voltage-gated channel subfamily A regulatory beta subunit 3	0.9	0.3	3.0	< 0.01
ENSSSCG00000026352	LOC100512369	protein unc-119 homolog A	8.9	3.0	2.9	< 0.01
ENSSSCG00000000893		amidohydrolase domain containing 1	7.6	2.6	2.9	< 0.01
ENSSSCG00000001767	LOC100153192		4.5	1.5	2.9	0.01
ENSSSCG00000007772		syntaxin 1B	2.5	0.9	2.9	< 0.01
ENSSSCG00000029435	LOC100626748	lysyl oxidase homolog 3	250.7	87.7	2.9	0.01
ENSSSCG00000015955	ITGA6	integrin subunit alpha 6	87.2	30.2	2.9	< 0.01
ENSSSCG00000016665	BMPER	BMP binding endothelial regulator	37.6	13.2	2.9	0.01
ENSSSCG00000023664	LOC100623790		27.7	9.6	2.9	< 0.01

Ensembl gene ID	Gene name	Gene description	C	Tx	FC	P
ENSSSCG00000000992		forkhead box F2	20.2	7.0	2.9	< 0.01
ENSSSCG00000027928	TMEM9	transmembrane protein 9	18.4	6.5	2.9	< 0.01
ENSSSCG00000000779	KIF21A	kinesin family member 21A	13.7	4.8	2.9	< 0.01
ENSSSCG00000017250	SLC39A11		5.4	1.9	2.9	< 0.01
ENSSSCG00000021558			3.8	1.3	2.9	0.01
ENSSSCG00000009862	RNFT2	ring finger protein, transmembrane 2	3.4	1.2	2.9	< 0.01
ENSSSCG00000011812	TPRG1	tumor protein p63 regulated 1	3.2	1.1	2.9	< 0.01
ENSSSCG00000005577	LOC100156463	zinc finger and BTB domain-containing protein 26	2.9	1.0	2.9	< 0.01
ENSSSCG00000003054	ZNF575	zinc finger protein 575	1.8	0.7	2.9	< 0.01
ENSSSCG00000028699	NEIL2	nei like DNA glycosylase 2	1.9	0.6	2.9	< 0.01
ENSSSCG00000006247	PLAG1	PLAG1 zinc finger	1.7	0.6	2.9	0.01
ENSSSCG00000014622	DNHD1	dynein heavy chain domain 1	0.3	0.1	2.9	0.01
ENSSSCG00000006857	COL11A1	collagen type XI alpha 1 chain	2585	927.3	2.8	0.01
ENSSSCG00000000848	GLT8D2	glycosyltransferase 8 domain containing 2	335.2	120.4	2.8	< 0.01
ENSSSCG00000030597	HAPLN3	hyaluronan and proteoglycan link protein 3	51.7	18.5	2.8	< 0.01
ENSSSCG00000024312	ID4	inhibitor of DNA binding 4, HLH protein	17.4	6.3	2.8	< 0.01
ENSSSCG00000001765	ADAMTS7	ADAM metallopeptidase with thrombospondin type 1 motif 7	5.8	2.1	2.8	< 0.01
ENSSSCG00000029353		zinc finger and SCAN domain containing 2	3.2	1.2	2.8	0.01
ENSSSCG00000009832	FAM109A	family with sequence similarity 109 member A	2.4	0.9	2.8	< 0.01
ENSSSCG00000022312	RHPN2	rhophilin Rho GTPase binding protein 2	2.2	0.8	2.8	< 0.01
ENSSSCG00000000117	LOC100737601		2.1	0.7	2.8	0.01
ENSSSCG00000023631			2.1	0.7	2.8	< 0.01
ENSSSCG00000008586	TP53I3	tumor protein p53 inducible protein 3	1.7	0.6	2.8	< 0.01
ENSSSCG00000025211	LOC102158157	protein phosphatase 1H-like	1.4	0.5	2.8	< 0.01
ENSSSCG00000022324	PPFIA3	PTPRF interacting protein alpha 3	1.4	0.5	2.8	< 0.01
ENSSSCG00000025557	LOC100625296	mediator of RNA polymerase II transcription subunit 25-like	1.1	0.4	2.8	0.01
ENSSSCG00000010099	LOC100510930		1.0	0.4	2.8	< 0.01
ENSSSCG00000007671	TFR2	transferrin receptor 2	0.4	0.2	2.8	0.01
ENSSSCG00000003439	DHRS3	dehydrogenase/reductase 3	206.4	76.2	2.7	< 0.01
ENSSSCG00000007872	XYLT1	xylosyltransferase 1	56.7	21.1	2.7	0.01

Ensembl gene ID	Gene name	Gene description	C	Tx	FC	P
ENSSSCG00000002621	TRAM2	translocation associated membrane protein 2	42.0	15.5	2.7	< 0.01
ENSSSCG00000022599	LOC100514845		13.4	4.9	2.7	< 0.01
ENSSSCG00000003848	LRP8	LDL receptor related protein 8	11.7	4.4	2.7	< 0.01
ENSSSCG00000010278	CDH23		6.1	2.3	2.7	0.01
ENSSSCG00000017328	ARHGAP27	Rho GTPase activating protein 27	5.8	2.1	2.7	< 0.01
ENSSSCG00000007344	KIAA1755	KIAA1755	3.8	1.4	2.7	< 0.01
ENSSSCG00000013266	LARGE2	LARGE xylosyl- and glucuronyltransferase 2	3.6	1.3	2.7	< 0.01
ENSSSCG00000017789	ABHD15	abhydrolase domain containing 15	3.6	1.3	2.7	< 0.01
ENSSSCG00000026089		cartilage acidic protein 1	2.7	1.0	2.7	< 0.01
ENSSSCG00000007052	FERMT1	fermitin family member 1	2.4	0.9	2.7	< 0.01
ENSSSCG00000007239	CCM2L	CCM2 like scaffolding protein	2.0	0.7	2.7	0.01
ENSSSCG00000021702	XRRA1		1.3	0.5	2.7	< 0.01
ENSSSCG00000017392	CCR10	C-C motif chemokine receptor 10	0.8	0.3	2.7	< 0.01
ENSSSCG00000008267	HTRA2		150.6	57.8	2.6	< 0.01
ENSSSCG00000025364	LOC102159675	uncharacterized LOC102159675	24.6	9.4	2.6	< 0.01
ENSSSCG00000006705			19.2	7.2	2.6	0.01
ENSSSCG00000024967		chromosome 7 open reading frame 61	13.9	5.3	2.6	< 0.01
ENSSSCG00000003113	C5AR2	complement component 5a receptor 2	12.2	4.6	2.6	< 0.01
ENSSSCG00000023399			11.9	4.5	2.6	0.01
ENSSSCG00000000277	NPFF	neuropeptide FF-amide peptide precursor	8.3	3.2	2.6	< 0.01
ENSSSCG00000000436	LOC100520306		6.0	2.3	2.6	< 0.01
ENSSSCG00000001411	APOM	Apolipoprotein M	2.8	1.1	2.6	0.01
ENSSSCG00000011624	MCM2	minichromosome maintenance complex component 2	2.4	0.9	2.6	< 0.01
ENSSSCG00000013718	LOC102166940	zinc finger protein 14-like	1.4	0.5	2.6	< 0.01
ENSSSCG00000022045			1.0	0.4	2.6	< 0.01
ENSSSCG00000000157	BPIFC	BPI fold containing family C	0.7	0.3	2.6	0.01
ENSSSCG00000017539	HOXB6	homeobox B6	20.1	7.8	2.6	< 0.01
ENSSSCG00000025620		prickle planar cell polarity protein 1	10.2	4.0	2.6	0.01
ENSSSCG00000002555	JAG2	jagged 2	9.0	3.5	2.6	0.01
ENSSSCG00000009373	SLC25A15	solute carrier family 25 member 2	7.5	2.9	2.6	0.01

Ensembl gene ID	Gene name	Gene description	C	Tx	FC	P
ENSSSCG00000009755	AACS	acetoacetyl-CoA synthetase	6.9	2.7	2.6	< 0.01
ENSSSCG00000030474			5.7	2.2	2.6	< 0.01
ENSSSCG00000028144	EPHX3	epoxide hydrolase 3	3.6	1.4	2.6	0.01
ENSSSCG00000022165			2.9	1.1	2.6	0.01
ENSSSCG00000013531			2.4	1.0	2.6	< 0.01
ENSSSCG00000003456			0.7	0.3	2.6	< 0.01
ENSSSCG00000030561	LMTK3	lemur tyrosine kinase 3	0.4	0.2	2.6	< 0.01
ENSSSCG00000012319	LOC100523107		87.0	35.1	2.5	< 0.01
ENSSSCG00000009930	GLTP	glycolipid transfer protein	40.3	16.3	2.5	< 0.01
ENSSSCG00000003755	MCOLN2	mucolipin 2	17.5	7.0	2.5	< 0.01
ENSSSCG00000023526		Rap guanine nucleotide exchange factor 3	16.1	6.4	2.5	< 0.01
ENSSSCG00000021515	HS3ST1	heparan sulfate-glucosamine 3-sulfotransferase 1	10.6	4.2	2.5	0.01
ENSSSCG00000015753	ANGPT2	angiopoietin 2	10.0	4.0	2.5	0.01
ENSSSCG00000003651	RHBDL2	rhomboid like 2	6.5	2.6	2.5	< 0.01
ENSSSCG00000002788	EXOC3L1	exocyst complex component 3 like 1	4.8	1.9	2.5	< 0.01
ENSSSCG00000027242			3.1	1.2	2.5	0.01
ENSSSCG00000013933	PBX4	PBX homeobox 4	0.7	0.3	2.5	< 0.01
ENSSSCG00000023762	TRPM2	transient receptor potential cation channel subfamily M member 2	0.6	0.2	2.5	< 0.01
ENSSSCG00000008006	FBXL16	F-box and leucine rich repeat protein 16	0.5	0.2	2.5	< 0.01
ENSSSCG00000004291	NT5E	5'-nucleotidase ecto	164.3	67.4	2.4	< 0.01
ENSSSCG00000003592	SDC3	syndecan 3	93.9	38.1	2.4	0.01
ENSSSCG00000008081	BARX1	BARX homeobox 1	40.8	16.9	2.4	< 0.01
ENSSSCG00000008281			39.7	16.3	2.4	< 0.01
ENSSSCG00000030472			34.2	14.1	2.4	< 0.01
ENSSSCG00000016548	PODXL	podocalyxin like	30.7	12.6	2.4	< 0.01
ENSSSCG00000014830	LOC100514530	cytochrome c oxidase assembly factor 4 homolog	26.4	10.7	2.4	< 0.01
ENSSSCG00000023783	LOC100737651	39S ribosomal protein L27, mitochondrial	16.9	6.9	2.4	0.01
ENSSSCG00000013049	RCOR2		12.1	4.9	2.4	< 0.01
ENSSSCG00000003421	FBXO44	F-box protein 44	11.0	4.5	2.4	0.01
ENSSSCG00000011618	GATA2	GATA binding protein 2	4.7	1.9	2.4	< 0.01

Ensembl gene ID	Gene name	Gene description	C	Tx	FC	P
ENSSSCG00000026550		zinc finger protein 621	3.9	1.6	2.4	< 0.01
ENSSSCG00000007765	PRSS53	protease, serine 53	2.8	1.1	2.4	< 0.01
ENSSSCG00000001847	MESP1	mesoderm posterior bHLH transcription factor 1	2.7	1.1	2.4	< 0.01
ENSSSCG00000025260	CARD10	caspase recruitment domain family member 10	2.4	1.0	2.4	0.01
ENSSSCG00000025488	MCM3	minichromosome maintenance complex component 3	2.1	0.9	2.4	< 0.01
ENSSSCG00000003238		sperm acrosome associated 6	2.0	0.8	2.4	< 0.01
ENSSSCG00000026689	CCDC114	coiled-coil domain containing 114	1.5	0.6	2.4	< 0.01
ENSSSCG00000013760	C2H19orf57	chromosome 19 open reading frame 57	0.5	0.2	2.4	< 0.01
ENSSSCG00000010581	PSD	pleckstrin and Sec7 domain containing	0.5	0.2	2.4	< 0.01
ENSSSCG00000028363	TMPRSS9	transmembrane protease, serine 9	0.4	0.2	2.4	< 0.01
ENSSSCG00000003549	LDLRAP1	low density lipoprotein receptor adaptor protein 1	20.0	8.4	2.4	< 0.01
ENSSSCG00000010698	FGFR2	fibroblast growth factor receptor 2 precursor	16.6	6.9	2.4	0.01
ENSSSCG00000001680	ABCC10		9.4	4.0	2.4	< 0.01
ENSSSCG00000010048	RAB36	RAB36, member RAS oncogene family	4.9	2.1	2.4	< 0.01
ENSSSCG00000001203	ZSCAN9	zinc finger and SCAN domain containing 9	3.6	1.5	2.4	0.01
ENSSSCG00000028381	FLYWCH1	FLYWCH-type zinc finger 1	3.0	1.3	2.4	< 0.01
ENSSSCG00000003233	ZNF175	zinc finger protein 175	2.2	0.9	2.4	0.01
ENSSSCG00000011683		procollagen C-endopeptidase enhancer 2	299.8	128.9	2.3	0.01
ENSSSCG00000015290			16.3	7.1	2.3	< 0.01
ENSSSCG00000014303	JADE2	jade family PHD finger 2	11.0	4.7	2.3	0.01
ENSSSCG00000022082			10.3	4.4	2.3	0.01
ENSSSCG00000013087	TKFC	triokinase and FMN cyclase	9.7	4.2	2.3	< 0.01
ENSSSCG00000000209	NCKAP5L	NCK associated protein 5 like	9.8	4.2	2.3	< 0.01
ENSSSCG00000009774	C14H12orf65	chromosome 12 open reading frame 65	9.0	3.8	2.3	< 0.01
ENSSSCG00000007454	ZMYND8	zinc finger MYND-type containing 8	7.6	3.2	2.3	0.01
ENSSSCG00000002783	SLC9A5	solute carrier family 9 member A5	7.5	3.2	2.3	< 0.01
ENSSSCG00000000066	L3MBTL2	L3MBTL2 polycomb repressive complex 1 subunit	5.5	2.4	2.3	< 0.01
ENSSSCG00000003340	CPTP	ceramide-1-phosphate transfer protein	4.1	1.8	2.3	0.01
ENSSSCG00000001637	GUCA1B	guanylate cyclase activator 1B	3.7	1.6	2.3	< 0.01
ENSSSCG00000007531	FAM217B	family with sequence similarity 217 member B	3.5	1.5	2.3	< 0.01

Ensembl gene ID	Gene name	Gene description	C	Tx	FC	P
ENSSSCG00000003287	BRSK1	BR serine/threonine kinase 1	2.7	1.1	2.3	< 0.01
ENSSSCG00000009901	SIRT4	sirtuin 4	2.4	1.0	2.3	< 0.01
ENSSSCG00000030432	RAB42	RAB42, member RAS oncogene family	2.3	1.0	2.3	< 0.01
ENSSSCG00000029909			1.2	0.5	2.3	0.01
ENSSSCG00000005050			1.1	0.5	2.3	0.01
ENSSSCG00000000398	APON		1.0	0.4	2.3	0.01
ENSSSCG00000023693	PROC	protein C, inactivator of coagulation factors Va and VIIIa	0.8	0.3	2.3	0.01
ENSSSCG00000009668	CLU	clusterin	2749	1197	2.3	< 0.01
ENSSSCG00000003681	TWSG1	twisted gastrulation BMP signaling modulator 1	194.7	84.9	2.3	< 0.01
ENSSSCG00000008101	FBLN7		160.1	70.5	2.3	0.01
ENSSSCG00000027550	PLCD1	phospholipase C delta 1	108.9	48.1	2.3	0.01
ENSSSCG00000017874	ATP2A3		13.2	5.8	2.3	< 0.01
ENSSSCG00000011392		family with sequence similarity 212 member A	11.7	5.1	2.3	< 0.01
ENSSSCG00000005593	OLFML2A	olfactomedin like 2A	11.3	5.0	2.3	0.01
ENSSSCG00000025105	LOC100523971		10.8	4.8	2.3	< 0.01
ENSSSCG00000017829		small G protein signaling modulator 2	9.7	4.3	2.3	< 0.01
ENSSSCG00000016763			9.5	4.2	2.3	< 0.01
ENSSSCG00000028443			8.3	3.6	2.3	< 0.01
ENSSSCG00000007385	KCNS1	potassium voltage-gated channel modifier subfamily S member 1	5.4	2.4	2.3	0.01
ENSSSCG00000014431	AFAP1L1	actin filament associated protein 1 like 1	4.7	2.1	2.3	0.01
ENSSSCG00000005152	IFN-DELTA-2	interferon-delta-2	4.3	1.9	2.3	< 0.01
ENSSSCG00000017972	LOC100523995	cytochrome b5 domain-containing protein 1	3.7	1.7	2.3	0.01
ENSSSCG00000010259	TYSND1	trypsin domain containing 1	3.5	1.5	2.3	0.01
ENSSSCG00000023933	CRACR2B	calcium release activated channel regulator 2B	2.4	1.1	2.3	0.01
ENSSSCG00000014897	FAM181B	family with sequence similarity 181 member B	2.3	1.0	2.3	0.01
ENSSSCG00000029138	LOC102161170	UDP-glucose:glycoprotein glucosyltransferase 1	2.2	1.0	2.3	< 0.01
ENSSSCG00000025351	ZBTB3	zinc finger and BTB domain containing 3	1.2	0.6	2.3	< 0.01
ENSSSCG00000006518	HCN3	hyperpolarization activated cyclic nucleotide gated potassium channel 3	0.7	0.3	2.3	< 0.01
ENSSSCG00000014876	MYO7A	unconventional myosin-VIIa	0.7	0.3	2.3	0.01
ENSSSCG00000029077	TUBAL3	tubulin alpha chain-like 3	0.3	0.1	2.3	0.01

Ensembl gene ID	Gene name	Gene description	C	Tx	FC	P
ENSSSCG00000000975	PANX2	pannexin 2	0.3	0.1	2.3	< 0.01
ENSSSCG00000024053	LOC100626097	fibrous sheath-interacting protein 2-like	0.2	0.1	2.3	0.01
ENSSSCG00000020915	P4HA2	prolyl 4-hydroxylase subunit alpha 2	299.4	134.7	2.2	0.01
ENSSSCG00000011813	P3H2	prolyl 3-hydroxylase 2	177.4	80.6	2.2	0.01
ENSSSCG00000025217	PYCR1	pyrroline-5-carboxylate reductase 1	176.5	79.2	2.2	< 0.01
ENSSSCG00000028814	SOD3	superoxide dismutase 3, extracellular	80.6	36.2	2.2	0.01
ENSSSCG00000016585	IMPDH1	inosine monophosphate dehydrogenase 1	73.4	32.8	2.2	< 0.01
ENSSSCG00000007112	PAX1	paired box 1	70.6	31.6	2.2	0.01
ENSSSCG00000003040	LOC100518073	Rho guanine nucleotide exchange factor 1	58.1	26.0	2.2	< 0.01
ENSSSCG00000023662	CHST3	carbohydrate sulfotransferase 3	57.3	25.5	2.2	0.01
ENSSSCG00000009138	CFI	complement factor I	31.2	14.2	2.2	0.01
ENSSSCG00000011592	PLXND1	plexin D1	16.8	7.6	2.2	< 0.01
ENSSSCG00000010746	ADAM12	ADAM metallopeptidase domain 12	14.1	6.3	2.2	0.01
ENSSSCG00000009822	FAM216A	family with sequence similarity 216 member A	9.6	4.4	2.2	< 0.01
ENSSSCG00000009807	RHOF	ras homolog family member F, filopodia associated	8.4	3.7	2.2	< 0.01
ENSSSCG00000027475			7.9	3.6	2.2	0.01
ENSSSCG00000001204	ZKSCAN4	zinc finger with KRAB and SCAN domains 4	2.6	1.2	2.2	0.01
ENSSSCG00000001822	RCCD1	RCC1 domain containing 1	1.4	0.6	2.2	< 0.01
ENSSSCG00000026564	CCDC169	coiled-coil domain containing 169	1.2	0.5	2.2	0.01
ENSSSCG00000003345	TMEM88B	transmembrane protein 88B	0.9	0.4	2.2	0.01
ENSSSCG00000011030			0.5	0.2	2.2	< 0.01
ENSSSCG00000001834	MFGE8	milk fat globule-EGF factor 8 protein	793.4	362.7	2.2	< 0.01
ENSSSCG00000010703	HTRA1	HtrA serine peptidase 1	531.1	243.3	2.2	< 0.01
ENSSSCG00000023863	LOC100739376	TSC22 domain family protein 1	182.1	84.4	2.2	0.01
ENSSSCG00000010527		MARVEL domain containing 1	120.5	56.0	2.2	< 0.01
ENSSSCG00000007116	CD93	CD93 molecule	40.5	18.7	2.2	< 0.01
ENSSSCG00000016436		chondroitin polymerizing factor 2	35.0	16.0	2.2	< 0.01
ENSSSCG00000022265	C2H19orf25	chromosome 19 open reading frame 25	22.0	10.1	2.2	< 0.01
ENSSSCG00000014035	B4GALT7	beta-1,4-galactosyltransferase 7	20.1	9.2	2.2	< 0.01
ENSSSCG00000008171	NPAS2	neuronal PAS domain protein 2	18.4	8.4	2.2	0.01

Ensembl gene ID	Gene name	Gene description	C	Tx	FC	P
ENSSSCG00000011699	HPS3	HPS3, biogenesis of lysosomal organelles complex 2 subunit 1	18.2	8.4	2.2	0.01
ENSSSCG00000009402		lysophosphatidic acid receptor 6	16.7	7.7	2.2	0.01
ENSSSCG00000009616	HR	hair growth associated	16.0	7.3	2.2	< 0.01
ENSSSCG00000022786	MOGS		12.0	5.5	2.2	< 0.01
ENSSSCG00000001440	NOTCH4	notch 4	9.5	4.4	2.2	< 0.01
ENSSSCG00000013619	SWSAP1	SWIM-type zinc finger 7 associated protein 1	9.1	4.2	2.2	0.01
ENSSSCG00000017635	MKS1	Meckel syndrome, type 1	8.1	3.7	2.2	< 0.01
ENSSSCG00000008545	ZNF512	zinc finger protein 512	7.3	3.4	2.2	0.01
ENSSSCG00000013882	FAM129C	family with sequence similarity 129 member C	5.5	2.5	2.2	0.01
ENSSSCG00000013858	CALR3	calreticulin 3	5.4	2.5	2.2	< 0.01
ENSSSCG00000004089	RMND1	required for meiotic nuclear division 1 homolog	5.3	2.4	2.2	< 0.01
ENSSSCG00000017518	OSBPL7	oxysterol binding protein like 7	4.9	2.3	2.2	0.01
ENSSSCG00000002786	ELMO3	engulfment and cell motility 3	4.6	2.2	2.2	< 0.01
ENSSSCG00000007712	DNAJC30	DnaJ heat shock protein family	3.7	1.7	2.2	0.01
ENSSSCG00000029165	DOK4	docking protein 4	3.7	1.7	2.2	< 0.01
ENSSSCG00000030363	LGI3	leucine rich repeat LGI family member 3	3.3	1.5	2.2	< 0.01
ENSSSCG00000007670	ACTL6B	actin like 6B	2.9	1.3	2.2	0.01
ENSSSCG00000028725	LOC100623827	transmembrane protein 102	2.8	1.3	2.2	0.01
ENSSSCG00000014892	USP35		2.6	1.2	2.2	0.01
ENSSSCG00000014034	N4BP3	NEDD4 binding protein 3	2.2	1.0	2.2	< 0.01
ENSSSCG00000006101	RAD54B	RAD54 homolog B	1.1	0.5	2.2	< 0.01
ENSSSCG00000003146	LOC100524377	netrin 5	0.7	0.3	2.2	< 0.01
ENSSSCG00000021250	FKBP9	FK506 binding protein 9	237.5	112.5	2.1	< 0.01
ENSSSCG00000011670	PXYLP1	2-phosphoxylose phosphatase 1	143.2	67.9	2.1	0.01
ENSSSCG00000007291	EDEM2	ER degradation enhancing alpha-mannosidase like protein 2	50.7	23.7	2.1	0.01
ENSSSCG00000006688	ANKRD35	ankyrin repeat domain 35	23.5	10.9	2.1	< 0.01
ENSSSCG00000025164	LOC100512339	TSC22 domain family protein 4	21.1	9.9	2.1	< 0.01
ENSSSCG00000017955	MPDU1		15.5	7.3	2.1	< 0.01
ENSSSCG00000027881	KCNK6	potassium two pore domain channel subfamily K member 6	14.7	6.9	2.1	0.01
ENSSSCG00000009071	JADE1	jade family PHD finger 1	10.9	5.2	2.1	< 0.01

Ensembl gene ID	Gene name	Gene description	C	Tx	FC	P
ENSSSCG00000017876	LOC100520452	cytochrome b5 domain containing 2	10.7	5.0	2.1	< 0.01
ENSSSCG00000011346			6.7	3.1	2.1	< 0.01
ENSSSCG00000023968	TSSK3	testis specific serine kinase 3	5.9	2.8	2.1	< 0.01
ENSSSCG00000007920	EEF2KMT		4.9	2.3	2.1	< 0.01
ENSSSCG00000024691			3.9	1.8	2.1	< 0.01
ENSSSCG00000004711	LCMT2	leucine carboxyl methyltransferase 2	3.8	1.8	2.1	0.01
ENSSSCG00000029812			3.2	1.5	2.1	0.01
ENSSSCG00000015277		SRY-box 13	2.6	1.2	2.1	0.01
ENSSSCG00000017602	LOC100739655		2.1	1.0	2.1	< 0.01
ENSSSCG00000008114			1.5	0.7	2.1	0.01
ENSSSCG00000025067	ECE2	endothelin converting enzyme 2	0.4	0.2	2.1	0.01
ENSSSCG00000001475	SLC39A7		141.7	68.7	2.1	< 0.01
ENSSSCG00000011787	MAGEF1	MAGE family member F1	24.6	11.7	2.1	< 0.01
ENSSSCG00000017531	LOC100523276		17.8	8.6	2.1	< 0.01
ENSSSCG00000010854	TMEM63A	transmembrane protein 63A	15.1	7.3	2.1	< 0.01
ENSSSCG00000011333	FAM212A	death domain-containing membrane protein NRADD	14.6	6.9	2.1	0.01
ENSSSCG00000001681	LOC100736948		13.1	6.3	2.1	< 0.01
ENSSSCG00000013595	CERS4	ceramide synthase 4	12.7	6.1	2.1	0.01
ENSSSCG00000000370	DGKA	Diacylglycerol kinase alpha	10.0	4.8	2.1	< 0.01
ENSSSCG00000004948	SMAD6	SMAD family member 6	9.6	4.6	2.1	< 0.01
ENSSSCG00000023283	LOC100621058		7.6	3.7	2.1	< 0.01
ENSSSCG00000005900	GPT	glutamic--pyruvic transaminase	7.6	3.7	2.1	< 0.01
ENSSSCG00000000268	AAAS	aladin WD repeat nucleoporin	5.1	2.4	2.1	< 0.01
ENSSSCG00000027478	PPCDC	phosphopantothenoylcysteine decarboxylase	4.6	2.2	2.1	0.01
ENSSSCG00000030596	HES2	hes family bHLH transcription factor 2	3.9	1.9	2.1	< 0.01
ENSSSCG00000021704	LOC100513144	protein tyrosine phosphatase domain-containing protein 1	3.6	1.7	2.1	< 0.01
ENSSSCG00000022280	DACT3	dishevelled binding antagonist of beta catenin 3	2.6	1.3	2.1	< 0.01
ENSSSCG00000002635	SPIRE2	spire type actin nucleation factor 2	1.6	0.8	2.1	0.01
ENSSSCG00000017540	HOXB5	homeobox B5	1.5	0.7	2.1	0.01
ENSSSCG00000013497	ANKRD24	ankyrin repeat domain 24	1.3	0.6	2.1	< 0.01

Ensembl gene ID	Gene name	Gene description	C	Tx	FC	P
ENSSSCG00000024000	LOC100738889	EF-hand and coiled-coil domain containing 1	0.9	0.4	2.1	0.01
ENSSSCG00000012766	BGN	biglycan	14796	7176	2.0	< 0.01
ENSSSCG00000029143	LOC100737666	collagen alpha-1 (XVI) chain	828.7	408.9	2.0	0.01
ENSSSCG00000011403	NAT6	N-acetyltransferase 6	50.4	24.5	2.0	< 0.01
ENSSSCG00000008313	LOC100739240	dysferlin-like	37.8	18.6	2.0	0.01
ENSSSCG00000006197	SULF1	sulfatase 1	26.3	12.9	2.0	0.01
ENSSSCG00000012350	MAGEH1	MAGE family member H1	25.2	12.4	2.0	< 0.01
ENSSSCG00000004622	GNB5	G protein subunit beta 5	22.8	11.2	2.0	< 0.01
ENSSSCG00000026994	ADAMTS10	ADAM metallopeptidase with thrombospondin type 1 motif 10	22.5	11.0	2.0	< 0.01
ENSSSCG00000025126	LGI4	leucine rich repeat LGI family member 4	21.6	10.7	2.0	0.01
ENSSSCG00000026654	LOC100153934	GDP-fucose protein O-fucosyltransferase 2-like	8.5	4.2	2.0	< 0.01
ENSSSCG00000010797	EARS2	glutamyl-tRNA synthetase 2, mitochondrial	7.0	3.4	2.0	0.01
ENSSSCG00000026507	LOC102158049	sarcoplasmic/endoplasmic reticulum calcium ATPase 3-like	6.4	3.2	2.0	< 0.01
ENSSSCG00000021740	METTL21B	methyltransferase like 21B	5.4	2.6	2.0	0.01
ENSSSCG00000027489	TMCO4	transmembrane and coiled-coil domains 4	4.9	2.4	2.0	< 0.01
ENSSSCG00000005896	LRRC24	leucine rich repeat containing 24	3.8	1.9	2.0	< 0.01
ENSSSCG00000027207	GPATCH2	G-patch domain containing 2	3.1	1.5	2.0	0.01
ENSSSCG00000009438	LOC100154105	kelch repeat and BTB domain containing 6	2.5	1.2	2.0	0.01
ENSSSCG00000003097	MYPOP	Myb related transcription factor, partner of profilin	1.8	0.9	2.0	< 0.01
ENSSSCG00000030818			1.1	0.5	2.0	< 0.01
ENSSSCG00000022975	AMT	aminomethyltransferase	0.4	0.2	2.0	0.01
ENSSSCG00000027442	TSC22D4	TSC22 domain family member 4	52.3	26.2	2.0	< 0.01
ENSSSCG00000016520	CREB3L2	cAMP responsive element binding protein 3 like 2	51.6	25.6	2.0	0.01
ENSSSCG00000029303			47.7	23.9	2.0	< 0.01
ENSSSCG00000000039	POLDIP3	DNA polymerase delta interacting protein 3	35.7	18.0	2.0	0.01
ENSSSCG00000021974			34.7	17.5	2.0	< 0.01
ENSSSCG00000023292		inverted formin, FH2 and WH2 domain containing	31.6	15.8	2.0	< 0.01
ENSSSCG00000011404	HYAL1	hyaluronidase-1 precursor	27.9	13.8	2.0	< 0.01
ENSSSCG00000005655	WDR34	WD repeat domain 34	18.5	9.2	2.0	< 0.01
ENSSSCG00000015819	PLPP5	phospholipid phosphatase 5	12.4	6.1	2.0	< 0.01

Ensembl gene ID	Gene name	Gene description	C	Tx	FC	P
ENSSSCG00000016753	POLD2	DNA polymerase delta 2, accessory subunit	9.7	4.8	2.0	0.01
ENSSSCG00000018026			7.7	3.8	2.0	< 0.01
ENSSSCG00000027986	ABCB8	ATP binding cassette subfamily B member 8	7.1	3.6	2.0	0.01
ENSSSCG00000001885	C7H15orf39	chromosome 15 open reading frame 39	5.4	2.7	2.0	< 0.01
ENSSSCG00000001206	ZSCAN26	zinc finger and SCAN domain containing 26	4.4	2.2	2.0	0.01
ENSSSCG00000008055	C3H16orf59	chromosome 16 open reading frame 59	4.4	2.2	2.0	< 0.01
ENSSSCG00000022672	TRPM4		3.9	2.0	2.0	0.01
ENSSSCG00000009055		ELMO domain containing 2	3.6	1.8	2.0	< 0.01
ENSSSCG00000030281	ZNF793	zinc finger protein 793	2.9	1.5	2.0	0.01
ENSSSCG00000030351	MEF2B	myocyte enhancer factor 2B	2.1	1.0	2.0	< 0.01
ENSSSCG00000028249	SKA3	spindle and kinetochore associated complex subunit 3	1.9	1.0	2.0	< 0.01
