# Supplementary material for: Repetitive in vivo manual loading of the spine elicits cellular responses in porcine annuli fibrosi
Source: PLoS One. 2021 Mar 23;16(3):e0248104. doi: 10.1371/journal.pone.0248104 (PMC7987143; doi:10.1371/journal.pone.0248104)
Supplement: S2 Table — (PDF) [file pone.0248104.s002.pdf]

Ensembl gene ID	Gene name	Gene description	Ctrl	Tx	FC	P
ENSSSCG00000022925	SLC2A3	solute carrier family 2 member 3á	73.9	262.7	3.6	0.01
ENSSSCG00000025889	LOC100739817	rho GTPase-activating protein 15-like	0.4	0.8	2.0	0.01
ENSSSCG00000021503	BCHE	butyrylcholinesterase	2.3	11.1	4.9	0.01
ENSSSCG00000012026	ADAMTS1	A disintegrin and metalloproteinase with thrombospondin motifs 1 precursor	10.2	49.7	4.9	0.01
ENSSSCG00000005426	FSD1L	fibronectin type III and SPRY domain containing 1 like	0.4	1.3	3.4	0.01
ENSSSCG00000004974	LARP	La ribonucleoprotein domain family member 6	14.6	48.8	3.4	0.01
ENSSSCG00000028986			1.2	3.5	3.0	0.01
ENSSSCG00000009592	NFIL3	nuclear factor, interleukin 3 regulated	41.5	98.0	2.4	0.01
ENSSSCG00000001478	BMP5	bone morphogenetic protein 5	2.0	8.1	4.0	0.01
ENSSSCG00000016957	CD180	CD180 molecule	0.2	0.4	2.8	0.01
ENSSSCG00000021651	SCN2B	sodium voltage-gated channel beta subunit 2	1.0	3.3	3.3	0.01
ENSSSCG00000016064	TMEFF2	transmembrane protein with EGF like and two follistatin like domains 2	4.5	29.1	6.5	0.01
ENSSSCG00000026454	PMAIP1	phorbol-12-myristate-13-acetate-induced protein 1	2.0	6.8	3.4	0.01
ENSSSCG00000010142	RYR2	ryanodine receptor 2	0.5	1.1	2.4	0.01
ENSSSCG00000010966	CCL19	chemokine	24.3	57.1	2.4	0.01
ENSSSCG00000006359	ADAMTS4	ADAM metallopeptidase with thrombospondin type 1 motif 4	6.2	65.4	10.6	0.01
ENSSSCG00000025049	CYBRD1	cytochrome b reductase 1	61.1	123.9	2.0	0.01
ENSSSCG00000026139	TIFA	TRAF interacting protein with forkhead associated domain	2.4	9.1	3.9	0.01
ENSSSCG00000009825	PPTC7	PTC7 protein phosphatase homolog	6.5	17.5	2.7	0.01
ENSSSCG00000000647	OLR1	oxidized low density lipoprotein	0.8	2.1	2.7	0.01
ENSSSCG00000022246	ICOSLG	inducible T cell costimulator ligand	1.8	4.6	2.6	0.01
ENSSSCG00000006345	OLFML2B	olfactomedin like 2B	40.3	82.6	2.1	0.01
ENSSSCG00000015302	STEAP2	STEAP2 metalloreductase	2.0	5.8	2.8	0.01
ENSSSCG00000000857	IGF1	insulin like growth factor 1	7.2	36.7	5.1	0.01
ENSSSCG00000004990	LRFN5	leucine rich repeat and fibronectin type III domain containing 5	0.2	0.8	4.3	0.01
ENSSSCG00000015435	NAMPT	nicotinamide phosphoribosyltransferase	17.0	43.0	2.5	0.01
ENSSSCG00000015595	ATF3	activating transcription factor 3	7.4	66.0	8.9	0.01
ENSSSCG00000010201	RASGEF1A	RasGEF domain family member 1A	0.2	0.7	3.8	0.01
ENSSSCG00000012050	RCAN1	regulator of calcineurin 1	160.9	569.0	3.5	0.01
ENSSSCG00000000136	CSF2RB	colony stimulating factor 2 receptor beta common subunit	2.1	5.4	2.6	0.01

Ensembl gene ID	Gene name	Gene description	Ctrl	Tx	FC	P
ENSSSCG00000006723			6.8	15.2	2.2	0.01
ENSSSCG00000012754	GABRE	gamma-aminobutyric acid type A receptor epsilon subunit	0.9	4.6	5.2	0.01
ENSSSCG00000016452	GIMAP8	GTPase, IMAP family member 8 [Source:HGNC Symbol;Acc:HGNC:21792]	0.2	1.0	5.6	0.01
ENSSSCG00000026364			0.9	2.8	3.1	0.01
ENSSSCG00000022961	CLMP	CXADR like membrane protein	16.9	39.7	2.4	0.01
ENSSSCG00000026249			64.2	153.2	2.4	0.01
ENSSSCG00000000951	CSRP2	cysteine and glycine rich protein 2	19.5	39.7	2.0	0.01
ENSSSCG00000022292	NPL	N-acetylneuraminate pyruvate lyase	1.0	2.5	2.6	0.01
ENSSSCG00000007682	SH2B2	SH2B adaptor protein 2	2.2	5.5	2.5	0.01
ENSSSCG00000008773	PGM2	phosphoglucomutase 2	11.6	24.3	2.1	0.01
ENSSSCG00000016009	ITGA4	integrin subunit alpha 4	0.8	3.3	4.4	0.01
ENSSSCG00000012571	COL4A6	collagen type IV alpha 6 chain	1.9	6.7	3.6	0.01
ENSSSCG00000003839	PLPP3	phospholipid phosphatase 3	178.3	420.2	2.4	0.01
ENSSSCG00000029488	LOC100738012	A disintegrin and metalloproteinase with thrombospondin motifs 1	15.6	64.8	4.2	0.01
ENSSSCG00000003558	RPS6KA1	ribosomal protein S6 kinase, 90kDa, polypeptide 1	1.7	3.8	2.2	0.01
ENSSSCG00000006053	BAALC	brain and acute leukemia cytoplasmic protein	7.5	19.1	2.5	0.01
ENSSSCG00000029691		coronin 1A	0.9	2.9	3.2	0.01
ENSSSCG00000004125	STX11	syntaxin 11	3.6	18.1	5.0	0.01
ENSSSCG00000006736	CD2	CD2 molecule	0.2	0.6	3.4	0.01
ENSSSCG00000011524	CHL1	cell adhesion molecule L1 like	0.6	1.6	2.9	0.01
ENSSSCG00000007395	SYS1	Sys1 golgi trafficking protein	19.0	40.6	2.1	0.01
ENSSSCG00000027602	PLBD1	phospholipase B domain containing 1	2.4	14.7	6.0	0.01
ENSSSCG00000004199	EPB41L2	erythrocyte membrane protein band 4.1 like 2	31.9	64.6	2.0	0.01
ENSSSCG00000006172	PI15	peptidase inhibitor 15	3.3	27.5	8.3	0.01
ENSSSCG00000010312	PLAU-201	plasminogen activator, urokinase	12.2	52.8	4.3	0.01
ENSSSCG00000030385		complement C3 precursor	1.6	10.2	6.4	0.01
ENSSSCG00000021862	PTGER2	prostaglandin E receptor 2	2.3	6.8	3.0	0.01
ENSSSCG00000006729	FAM46C	family with sequence similarity 46 member C	1.2	3.3	2.7	0.01
ENSSSCG00000012631	LONRF3	LON peptidase N-terminal domain and ring finger 3	15.7	36.3	2.3	0.01
ENSSSCG00000028760			4.4	13.8	3.1	0.01

Ensembl gene ID	Gene name	Gene description	Ctrl	Tx	FC	P
ENSSSCG00000021967			0.4	0.9	2.4	0.01
ENSSSCG00000010381	ARHGAP22	Rho GTPase activating protein 22á	1.0	2.4	2.4	0.01
ENSSSCG00000024190			1.4	3.2	2.2	0.01
ENSSSCG00000021620	STIP1	stress induced phosphoprotein 1	33.8	69.8	2.1	0.01
ENSSSCG00000012395	NLGN3		0.2	0.9	3.8	0.01
ENSSSCG00000023103	LOC100624711	metalloreductase STEAP2	3.6	11.6	3.2	0.01
ENSSSCG00000015025	CRYAB	crystallin alpha B	106.8	454.6	4.3	0.01
ENSSSCG00000000130	CYTH	cytohesin 4	1.1	3.5	3.3	0.01
ENSSSCG00000029927			0.8	1.9	2.5	0.01
ENSSSCG00000008769	LOC100520753	macrosialin	0.6	1.6	2.8	0.01
ENSSSCG00000012029	BACH1	BTB domain and CNC homolog 1	5.7	14.0	2.5	0.01
ENSSSCG00000027549			0.7	4.1	5.6	0.01
ENSSSCG00000001572	FGD2	FYVE, RhoGEF and PH domain containing 2	0.7	3.2	4.7	0.01
ENSSSCG00000021409		tribbles pseudokinase 1	2.3	19.3	8.4	0.01
ENSSSCG00000010893	CFH	complement factor H precursor	5.2	42.0	8.1	0.01
ENSSSCG00000011110		coiled-coil domain containing 3	13.8	58.4	4.2	0.01
ENSSSCG00000021474			1.5	6.0	4.0	0.01
ENSSSCG00000013614	CNN1	calponin 1	8.2	23.7	2.9	0.01
ENSSSCG00000026931	SERTAD1	SERTA domain containing 1	17.5	36.7	2.1	0.01
ENSSSCG00000014164			1.9	4.0	2.1	0.01
ENSSSCG00000013599	ANGPTL4	Angiopoietin-related protein 4	15.0	151.7	10.1	0.01
ENSSSCG00000012480	TNMD	tenomodulin	47.1	608.1	12.9	0.01
ENSSSCG00000025584			0.5	1.4	3.1	0.01
ENSSSCG00000024528		zinc finger DHHC-type containing 14	1.0	2.2	2.1	0.01
ENSSSCG00000006416	ACKR1	atypical chemokine receptor 1	5.3	73.1	13.8	0.01
ENSSSCG00000000456	SLC16A7	solute carrier family 16 member 7	0.8	3.4	4.6	0.01
ENSSSCG00000022254	LOC106507277	growth arrest-specific protein 2	14.2	45.8	3.2	0.01
ENSSSCG00000021386	PTGR1	prostaglandin reductase 1	9.5	23.4	2.5	0.01
ENSSSCG00000013546	LOC100737024	DENN domain containing 1C	0.3	0.7	2.8	0.01
ENSSSCG00000030582	BATF	basic leucine zipper transcription factor, ATF-like	2.1	5.0	2.4	0.01

Ensembl gene ID	Gene name	Gene description	Ctrl	Tx	FC	P
ENSSSCG00000008334	MXD1	MAX dimerization protein 1	4.2	8.7	2.1	0.01
ENSSSCG00000011038	MRC1	mannose receptor C-type 1	2.0	17.9	8.8	0.01
ENSSSCG00000000242	LOC396594	growth-regulated protein homolog gamma	1.7	13.0	7.6	0.01
ENSSSCG00000010959			3.3	12.4	3.7	0.01
ENSSSCG00000015617	G0S2	G0/G1 switch 2	2.1	7.5	3.5	0.01
ENSSSCG00000002727	IL34	interleukin 34	10.0	24.1	2.4	0.01
ENSSSCG00000030088		colony stimulating factor 2 receptor alpha subunit	0.7	3.3	4.7	0.01
ENSSSCG00000025399			3.0	13.7	4.5	0.01
ENSSSCG00000010383	WDFY4	WDFY family member 4	0.4	0.9	2.4	0.01
ENSSSCG00000029507	RASGEF1B	ras-GEF domain-containing family member 1B	3.5	12.1	3.5	0.01
ENSSSCG00000016083	PLCL1	phospholipase C like 1	2.3	5.3	2.3	0.01
ENSSSCG00000009664	PTK2B	protein tyrosine kinase 2 beta	0.6	2.9	5.1	0.01
ENSSSCG00000027272	PIK3CG	phosphatidylinositol-4,5-bisphosphate 3-kinase catalytic subunit gamma	0.4	1.4	3.2	0.01
ENSSSCG00000015884	CD302	CD302 molecule	15.0	37.1	2.5	0.01
ENSSSCG00000016396	TNFAIP6	tumor necrosis factor, alpha-induced protein 6	31.5	69.5	2.2	0.01
ENSSSCG00000011925	CD200R1	cell surface glycoprotein CD200 receptor 1 precursor	0.3	1.7	5.3	0.01
ENSSSCG00000017008	DOCK2	dedicator of cytokinesis 2	0.2	1.1	5.3	0.01
ENSSSCG00000005472	SLC46A2	solute carrier family 46 member 2	0.4	1.8	4.6	0.01
ENSSSCG00000024518	SIGLEC5	sialic acid binding Ig-like lectin 5	0.4	1.6	4.4	0.01
ENSSSCG00000017607	TMEM100	transmembrane protein 100	17.5	75.6	4.3	0.01
ENSSSCG00000029855	LHFPL6	LHFPL tetraspan subfamily member 6	170.0	484.5	2.9	0.01
ENSSSCG00000011647			1.6	4.2	2.6	0.01
ENSSSCG00000022103			0.4	0.8	2.1	0.01
ENSSSCG00000011322	CCR1	C-C motif chemokine receptor 1	0.8	6.8	8.6	0.01
ENSSSCG00000030498	LOC100625743	growth arrest-specific protein 2	9.1	32.0	3.5	0.01
ENSSSCG00000009240	LOC100525175	placenta specific 8	0.8	5.2	6.4	0.01
ENSSSCG00000022780	UGCG	UDP-glucose ceramide glucosyltransferase	2.8	9.5	3.4	0.01
ENSSSCG00000012126	GPM6B	glycoprotein M6B	4.3	13.4	3.1	0.01
ENSSSCG00000016033		GULP, engulfment adaptor PTB domain containing 1	9.5	19.5	2.1	0.01
ENSSSCG00000004307	akirin 2		7.5	15.4	2.1	0.01

Ensembl gene ID	Gene name	Gene description	Ctrl	Tx	FC	P
ENSSSCG00000030638	LOC100511343	platelet glycoprotein 4-like	1.0	6.3	6.1	0.01
ENSSSCG00000026123			6.2	22.8	3.7	0.01
ENSSSCG00000010509	PIK3AP1	phosphoinositide-3-kinase adaptor protein 1	1.0	3.4	3.6	0.01
ENSSSCG00000021933	CLEC5A	C-type lectin domain family 5 member A-like	0.4	3.5	9.8	0.01
ENSSSCG00000003928	PLK3	polo like kinase 3	4.0	9.5	2.4	0.01
ENSSSCG00000027941	LSAMP	limbic system-associated membrane protein	0.2	1.6	6.7	0.01
ENSSSCG00000010504	BLNK	B-cell linker	0.4	1.9	4.5	0.01
ENSSSCG00000006549			1.2	4.6	3.7	0.01
ENSSSCG00000028435			0.2	1.6	9.1	0.01
ENSSSCG00000007336	NNAT	neuronatin	2.7	10.2	3.8	0.01
ENSSSCG00000027824			1.2	4.5	3.8	0.01
ENSSSCG00000001582	MDGA1	MAM domain containing glycosylphosphatidylinositol anchor 1	0.3	1.1	3.6	0.01
ENSSSCG00000016589	LRRC4	leucine rich repeat containing 4	0.5	1.3	2.8	0.01
ENSSSCG00000020754	CD3EAP	CD3e molecule associated protein	5.7	12.2	2.1	0.01
ENSSSCG00000005965	MYC	myc proto-oncogene protein	9.6	40.2	4.2	0.01
ENSSSCG00000006648	CTSS	cathepsin S	21.6	51.3	2.4	0.01
ENSSSCG00000022689	GADD45B	growth arrest and DNA damage inducible beta	71.7	178.3	2.5	0.01
ENSSSCG00000029956			7.4	15.9	2.1	0.01
ENSSSCG00000000734			1.2	5.9	4.8	0.01
ENSSSCG00000013388			2.3	7.1	3.2	0.01
ENSSSCG00000022624	LOC100627373	coronin-1C-like	7.9	18.6	2.4	0.01
ENSSSCG00000027121	ODC1	ornithine decarboxylase	24.6	53.4	2.2	0.01
ENSSSCG00000021232	SYNC	syncoilin, intermediate filament protein	3.6	7.2	2.0	0.01
ENSSSCG00000023610			4.8	17.4	3.6	0.01
ENSSSCG00000013473	GNA15	G protein subunit alpha 15	0.5	1.8	3.6	0.01
ENSSSCG00000013144	MPEG1	macrophage expressed 1	4.9	14.9	3.0	0.01
ENSSSCG00000012068		ETS proto-oncogene 2, transcription factor	26.0	65.8	2.5	0.01
ENSSSCG00000021774	B3GALNT1	beta-1,3-N-acetylgalactosaminyltransferase 1 (globoside blood group)	2.3	8.3	3.6	< 0.01
ENSSSCG00000016573	IRF5	interferon regulatory factor 5	2.9	8.2	2.9	< 0.01
ENSSSCG00000030898	MAFB	MAF bZIP transcription factor B	11.9	29.0	2.5	< 0.01

Ensembl gene ID	Gene name	Gene description	Ctrl	Tx	FC	P
ENSSSCG00000002554	PLD4	phospholipase D family member 4	1.2	4.2	3.4	< 0.01
ENSSSCG00000003709	LOC106504078	laminin subunit alpha-3-like	3.0	6.4	2.1	< 0.01
ENSSSCG00000012397	IL2RG	interleukin 2 receptor subunit gamma	1.9	3.9	2.0	< 0.01
ENSSSCG00000030012		olfactomedin like 1	0.3	2.2	7.5	< 0.01
ENSSSCG00000016799		cadherin 12	2.3	7.8	3.5	< 0.01
ENSSSCG00000015301	LOC100739711	metalloreductase STEAP1	15.8	54.7	3.5	< 0.01
ENSSSCG00000024495	SELPLG-201	selectin P ligand	1.0	3.2	3.3	< 0.01
ENSSSCG00000013411	WEE1	WEE1 G2 checkpoint kinase	9.3	21.1	2.3	< 0.01
ENSSSCG00000028326			22.6	148.6	6.6	< 0.01
ENSSSCG00000028241	LOC100738358	V-set and immunoglobulin domain-containing protein 4	3.0	15.3	5.1	< 0.01
ENSSSCG00000016583	FAM71F2	family with sequence similarity 71 member F2	1.3	2.7	2.0	< 0.01
ENSSSCG00000029140	CCL24	C-C motif chemokine ligand 24	0.7	10.7	14.4	< 0.01
ENSSSCG00000010808	RGS18	regulator of G-protein signaling 18	0.3	2.0	7.3	< 0.01
ENSSSCG00000024596	NOCT	nocturnin	6.8	34.0	5.0	< 0.01
ENSSSCG00000025537	PTPN6	protein tyrosine phosphatase, non-receptor type 6	1.3	4.5	3.5	< 0.01
ENSSSCG00000015085	IL10RA	interleukin 10 receptor subunit alpha	2.0	5.6	2.8	< 0.01
ENSSSCG00000026618	CAVIN2	caveolae associated protein 2á	12.1	25.5	2.1	< 0.01
ENSSSCG00000011534	BHLHE40	basic helix-loop-helix family member e40	195.1	490.1	2.5	< 0.01
ENSSSCG00000008959	CXCL2	C-X-C motif chemokine 2 precursor	4.8	22.2	4.7	< 0.01
ENSSSCG00000008072	ASPN	asporin	47.4	187.1	3.9	< 0.01
ENSSSCG00000010908	PTPRC	protein tyrosine phosphatase, receptor type Cá	2.0	6.7	3.5	< 0.01
ENSSSCG00000027855	SOCS1	suppressor of cytokine signaling 1	3.5	7.6	2.2	< 0.01
ENSSSCG00000001456	SLA-DQA1	SLA class II histocompatibility antigen, DQ haplotype D alpha chain precursor	72.5	151.1	2.1	< 0.01
ENSSSCG00000015671	ARHGAP15	Rho GTPase activating protein 15	0.2	1.2	5.4	< 0.01
ENSSSCG00000015307		cyclin dependent kinase 14	2.7	6.1	2.3	< 0.01
ENSSSCG00000015404			28.5	59.4	2.1	< 0.01
ENSSSCG00000012121	EGFL6	EGF like domain multiple 6	5.0	32.0	6.4	< 0.01
ENSSSCG00000009630	EGR3	early growth response 3	0.3	1.7	5.1	< 0.01
ENSSSCG00000003528	LOC100739136	complement C1q subcomponent subunit B-like	28.7	104.3	3.6	< 0.01
ENSSSCG00000010629	ADRA2A	Alpha-2A adrenergic receptor	0.8	2.4	2.9	< 0.01

Ensembl gene ID	Gene name	Gene description	Ctrl	Tx	FC	P
ENSSSCG00000021206	IL1RAP	interleukin 1 receptor accessory protein	1.1	2.5	2.3	< 0.01
ENSSSCG00000002962	MAP4K1	mitogen-activated protein kinase kinase kinase kinase 1	0.2	0.9	3.6	< 0.01
ENSSSCG00000013554	TRIP10		31.2	83.0	2.7	< 0.01
ENSSSCG00000003275	LOC100515837	leukocyte-associated immunoglobulin-like receptor 1	0.9	5.5	5.8	< 0.01
ENSSSCG00000003236	SIGLEC5	sialic acid binding Ig like lectin 5	0.3	1.7	5.2	< 0.01
ENSSSCG00000009462	KLF5		4.7	15.5	3.3	< 0.01
ENSSSCG00000012315		synaptojanin 2	5.4	13.1	2.4	< 0.01
ENSSSCG00000000601	PTPRO	protein tyrosine phosphatase, receptor type O	0.1	0.9	17.0	< 0.01
ENSSSCG00000005443	CTNNAL1	catenin alpha like 1	3.5	11.9	3.4	< 0.01
ENSSSCG00000010404	ALOX5	arachidonate 5-lipoxygenase	0.7	2.5	3.3	< 0.01
ENSSSCG00000022304			12.1	40.3	3.3	< 0.01
ENSSSCG00000008833	SGCB	sarcoglycan beta	13.9	37.2	2.7	< 0.01
ENSSSCG00000020792			2.6	5.4	2.1	< 0.01
ENSSSCG00000012572			2.9	10.2	3.5	< 0.01
ENSSSCG00000010450	LIPA	lipase A, lysosomal acid type	5.6	14.2	2.6	< 0.01
ENSSSCG00000023420	LOC100739312	protein unc-93 homolog B1-like	0.2	1.1	4.5	< 0.01
ENSSSCG00000003524	C1QA	complement component 1, q subcomponent, A chain	23.6	99.2	4.2	< 0.01
ENSSSCG00000026608	HTRA4	HtrA serine peptidase 4	1.4	3.4	2.5	< 0.01
ENSSSCG00000020705	MAP3K8	mitogen-activated protein kinase kinase kinase 8	6.5	13.8	2.1	< 0.01
ENSSSCG00000012117	TLR7	toll-like receptor 7	0.4	1.3	3.1	< 0.01
ENSSSCG00000004361		glutamate ionotropic receptor kainate type subunit 2	0.2	0.6	2.7	< 0.01
ENSSSCG00000027348	WIPF1	WAS/WASL interacting protein family member 1	14.7	38.5	2.6	< 0.01
ENSSSCG00000003088	APOE	apolipoprotein E	48.7	110.2	2.3	< 0.01
ENSSSCG00000004938	DENND4A	DENN domain containing 4A	7.7	16.8	2.2	< 0.01
ENSSSCG00000007240	HCK	HCK proto-oncogene, Src family tyrosine kinase	0.3	1.8	6.3	< 0.01
ENSSSCG00000010705	GMFG	glia maturation factor gamma	4.3	18.5	4.3	< 0.01
ENSSSCG00000007058	PLCB4	phospholipase C beta 4	2.2	5.5	2.5	< 0.01
ENSSSCG00000022355		CD37 molecule	0.3	1.5	6.1	< 0.01
ENSSSCG00000015663	C4BPA	complement component 4 binding protein, alpha	28.1	169.7	6.1	< 0.01
ENSSSCG00000022309	GPR34	G protein-coupled receptor 34	3.5	14.0	4.0	< 0.01

Ensembl gene ID	Gene name	Gene description	Ctrl	Tx	FC	P
ENSSSCG00000025167			0.9	3.2	3.7	< 0.01
ENSSSCG00000009454	LOC100520265		0.6	2.1	3.6	< 0.01
ENSSSCG00000024595	APOBR	apolipoprotein B receptor	0.5	1.5	2.8	< 0.01
ENSSSCG00000006290	SLC19A2	solute carrier family 19 member 2	3.5	15.7	4.6	< 0.01
ENSSSCG00000006357	FCER1G	High affinity immunoglobulin epsilon receptor subunit gamma	10.4	35.2	3.4	< 0.01
ENSSSCG00000006759	OLFML3	olfactomedin like 3	21.2	47.7	2.3	< 0.01
ENSSSCG00000017721	CCL8	chemokine ligand 8	0.1	4.2	49.5	< 0.01
ENSSSCG00000017006	LCP2	lymphocyte cytosolic protein 2	0.6	3.1	4.9	< 0.01
ENSSSCG00000026842		cadherin 18	1.9	5.8	3.1	< 0.01
ENSSSCG00000006288	SELP	P-selectin precursor	0.8	6.0	7.8	< 0.01
ENSSSCG00000013940		NLR family pyrin domain containing 3	0.2	1.5	7.0	< 0.01
ENSSSCG00000023374	SRGN	serglycin	10.9	67.3	6.2	< 0.01
ENSSSCG00000017062	TIMD4	T-cell immunoglobulin and mucin domain containing 4á	0.3	1.2	4.6	< 0.01
ENSSSCG00000016451	GIMAP1	GTPase, IMAP family member 1	3.9	14.1	3.7	< 0.01
ENSSSCG00000002252	ARRDC4	arrestin domain containing 4	6.7	15.4	2.3	< 0.01
ENSSSCG00000027828			0.4	2.3	5.5	< 0.01
ENSSSCG00000028536	LHFPL2	lipoma HMGIC fusion partner-like 2	19.0	71.4	3.8	< 0.01
ENSSSCG00000015774		teneurin transmembrane protein 3	2.2	6.5	3.0	< 0.01
ENSSSCG00000002535	HSP90AA1	heat shock protein 90 alpha family class A member 1	223.4	568.3	2.5	< 0.01
ENSSSCG00000009334	HSPH1	heat shock protein 105 kDa	22.1	116.2	5.3	< 0.01
ENSSSCG00000014336	EGR1	early growth response 1	13.7	116.7	8.5	< 0.01
ENSSSCG00000010665	SHTN1	shootin 1	0.1	0.8	6.6	< 0.01
ENSSSCG00000017258	FAM20A	FAM20A, golgi associated secretory pathway pseudokinase	1.5	5.5	3.7	< 0.01
ENSSSCG00000025133	ITGB2	integrin subunit beta 2	2.1	5.7	2.7	< 0.01
ENSSSCG00000023184			2.0	5.1	2.6	< 0.01
ENSSSCG00000011713	P2RY12	purinergic receptor P2Y12	0.7	5.9	9.1	< 0.01
ENSSSCG00000012364			1.1	6.3	5.6	< 0.01
ENSSSCG00000012636			2.4	9.1	3.8	< 0.01
ENSSSCG00000004597	AQP9	aquaporin 9	0.4	1.4	3.5	< 0.01
ENSSSCG00000012173	SAT1	Diamine acetyltransferase 1	82.2	285.3	3.5	< 0.01

Ensembl gene ID	Gene name	Gene description	Ctrl	Tx	FC	P
ENSSSCG00000004829	CACHD1	cache domain containing 1	1.6	4.6	2.8	< 0.01
ENSSSCG00000003525	C1QC	complement component 1, q subcomponent, C chain	24.5	105.5	4.3	< 0.01
ENSSSCG00000002829	MMP2	72 kDa type IV collagenase precursor	171.0	424.9	2.5	< 0.01
ENSSSCG00000014934	CHORDC1	cysteine and histidine rich domain containing 1	6.0	12.7	2.1	< 0.01
ENSSSCG00000014441	CSF1R	colony stimulating factor 1 receptor	3.9	20.2	5.1	< 0.01
ENSSSCG00000009468	KCTD12	potassium channel tetramerization domain containing 12	1.6	6.5	4.0	< 0.01
ENSSSCG00000029371	C5AR1	complement component 5a receptor 1	3.7	14.0	3.7	< 0.01
ENSSSCG00000011000	DNAJA1	DnaJ heat shock protein family member A1	46.4	123.9	2.7	< 0.01
ENSSSCG00000000675	LOC445464	complement C1r	39.1	91.3	2.3	< 0.01
ENSSSCG00000027777	PRRG1	proline rich and Gla domain 1	3.6	7.5	2.1	< 0.01
ENSSSCG00000017723	CCL2	C-C motif chemokine 2	5.6	146.2	26.3	< 0.01
ENSSSCG00000025686	KMO	kynurenine 3-monooxygenase	0.5	1.9	4.2	< 0.01
ENSSSCG00000015567	FAM129A	protein Niban-like	7.4	21.8	2.9	< 0.01
ENSSSCG00000021208			136.8	347.2	2.5	< 0.01
ENSSSCG00000011877	CD86	CD86 molecule	1.9	4.4	2.3	< 0.01
ENSSSCG00000013775	ADGRE5	adhesion G protein-coupled receptor E5	15.6	31.1	2.0	< 0.01
ENSSSCG00000025390	LOC100624077	endogenous retrovirus group V member 2 Env polyprotein	4.5	60.7	13.5	< 0.01
ENSSSCG00000013418	CFD	complement factor D	9.9	52.0	5.3	< 0.01
ENSSSCG00000029231	LOC100517099	leukocyte immunoglobulin-like receptor subfamily B member 3	1.0	3.4	3.3	< 0.01
ENSSSCG00000005225	RFX3	regulatory factor X3	0.9	1.8	2.0	< 0.01
ENSSSCG00000024310	LOC100153504	coagulation factor XIII A chain	2.8	9.8	3.5	< 0.01
ENSSSCG00000006372	ARHGAP30	Rho GTPase activating protein 30	0.9	2.8	3.2	< 0.01
ENSSSCG00000028137			11.8	31.3	2.7	< 0.01
ENSSSCG00000009997	OSM	oncostatin M	0.9	2.3	2.6	< 0.01
ENSSSCG00000027701	RP2	retinitis pigmentosa 2 (x-linked recessive)	4.2	9.3	2.3	< 0.01
ENSSSCG00000013551			0.5	3.4	7.6	< 0.01
ENSSSCG00000023868	C3AR1	complement C3a receptor 1	1.2	6.5	5.5	< 0.01
ENSSSCG00000000295			1.2	5.0	4.1	< 0.01
ENSSSCG00000001494	BAG2	BCL2 associated athanogene 2	8.9	28.0	3.1	< 0.01
ENSSSCG00000010204	BICC1	BicC family RNA binding protein 1	5.5	12.9	2.3	< 0.01

Ensembl gene ID	Gene name	Gene description	Ctrl	Tx	FC	P
ENSSSCG00000030379	YPEL1	yippee like 1	3.2	7.0	2.2	< 0.01
ENSSSCG00000006286	SELE	selectin E	1.5	11.5	7.6	< 0.01
ENSSSCG00000028711	CASP1	caspase-1	0.3	1.8	6.2	< 0.01
ENSSSCG00000001975	PRKD1	protein kinase D1	7.8	16.1	2.1	< 0.01
ENSSSCG00000022427	LOC397336	regulator of G-protein signaling 1	2.2	11.8	5.5	< 0.01
ENSSSCG00000011436	TLR9	Toll-like receptor 9	0.1	0.3	5.3	< 0.01
ENSSSCG00000027826			2.4	12.8	5.3	< 0.01
ENSSSCG00000003526	C1QB		11.1	51.2	4.6	< 0.01
ENSSSCG00000011264	CSRNP1	cysteine and serine rich nuclear protein 1	9.5	32.6	3.4	< 0.01
ENSSSCG00000001408	AIF1	allograft inflammatory factor 1	6.3	18.3	2.9	< 0.01
ENSSSCG00000015140			230.0	629.3	2.7	< 0.01
ENSSSCG00000004136			24.1	52.5	2.2	< 0.01
ENSSSCG00000005385	NR4A3	nuclear receptor subfamily 4 group A member 3	1.6	10.8	6.9	< 0.01
ENSSSCG00000011999	CADM2		1.0	4.1	3.9	< 0.01
ENSSSCG00000014277	IRF1	Interferon regulatory factor 1	9.5	34.5	3.6	< 0.01
ENSSSCG00000012637			3.7	12.9	3.5	< 0.01
ENSSSCG00000010772	ADAM8	ADAM metallopeptidase domain 8	2.4	7.8	3.3	< 0.01
ENSSSCG00000001457	SLA-DQB1-001	major histocompatibility complex, class II, DQ beta 2	55.9	124.5	2.2	< 0.01
ENSSSCG00000006979	MSR1	macrophage scavenger receptor 1	3.0	22.9	7.8	< 0.01
ENSSSCG00000006800	CD53	CD53 molecule	6.0	40.6	6.8	< 0.01
ENSSSCG00000000907		plexin C1	0.7	2.4	3.6	< 0.01
ENSSSCG00000016637	TFEC	transcription factor EC	0.2	1.5	6.6	< 0.01
ENSSSCG00000010892	KCNT2	potassium sodium-activated channel subfamily T member 2	0.5	1.9	3.9	< 0.01
ENSSSCG00000013604	MYO1F	myosin IF	1.3	3.6	2.8	< 0.01
ENSSSCG00000009412	LCP1	lymphocyte cytosolic protein 1	3.4	18.4	5.4	< 0.01
ENSSSCG00000014310	CXCL14	C-X-C motif chemokine ligand 14	5.3	24.0	4.5	< 0.01
ENSSSCG00000017956	LOC103158530	macrosialin	8.6	29.6	3.4	< 0.01
ENSSSCG00000013655	ICAM1	intercellular adhesion molecule 1	7.4	22.9	3.1	< 0.01
ENSSSCG00000005457		lysophosphatidic acid receptor 1	21.5	55.8	2.6	< 0.01
ENSSSCG00000009002	TLR2	toll like receptor 2	11.3	28.9	2.6	< 0.01

Ensembl gene ID	Gene name	Gene description	Ctrl	Tx	FC	P
ENSSSCG00000002919	TYROBP	TYRO protein tyrosine kinase binding protein	12.0	56.8	4.7	< 0.01
ENSSSCG00000017750		ecotropic viral integration site 2B	1.1	3.9	3.6	< 0.01
ENSSSCG00000023108	LOC100737924	essential MCU regulator, mitochondrial-like	5.6	14.7	2.6	< 0.01
ENSSSCG00000020649	LOC100620419	ADP-ribosylation factor-like protein 4C	1.2	2.9	2.4	< 0.01
ENSSSCG00000023942	ARL4C	ADP ribosylation factor like GTPase 4C	1.2	2.9	2.4	< 0.01
ENSSSCG00000029226	UBC	ubiquitin C	1544.4	3158.8	2.1	< 0.01
ENSSSCG00000030143	OGN	osteoglycin	119.2	494.2	4.2	< 0.01
ENSSSCG00000030092	LOC100624689	band 4.1-like protein 2	2.1	7.3	3.6	< 0.01
ENSSSCG00000016991	DUSP1	dual specificity phosphatase 1	134.5	337.2	2.5	< 0.01
ENSSSCG00000030156	APBB1IP	amyloid beta precursor protein binding family B member 1 interacting protein	0.3	2.2	6.5	< 0.01
ENSSSCG00000010708	ZFP36-001		67.4	298.8	4.4	< 0.01
ENSSSCG00000003766	(DNAJB4	DnaJ heat shock protein family B4	28.6	77.2	2.7	< 0.01
ENSSSCG00000006893	BCAR3	breast cancer anti-estrogen resistance 3	1.4	3.7	2.7	< 0.01
ENSSSCG00000005450		paralemmin 2	0.8	1.7	2.2	< 0.01
ENSSSCG00000004053	TAGAP	t-cell activation Rho GTPase-activating protein	1.1	2.4	2.2	< 0.01
ENSSSCG00000016076	COQ10B	coenzyme Q10B	10.2	21.1	2.1	< 0.01
ENSSSCG00000017700	CCL3L1		0.5	5.5	10.8	< 0.01
ENSSSCG00000008074	LOC10216701	uncharacterized LOC102167011	80.5	355.7	4.4	< 0.01
ENSSSCG00000011951	NFKBIZ	NFKB inhibitor zeta	11.2	33.5	3.0	< 0.01
ENSSSCG00000013041	FERMT3	fermitin family member 3	1.7	4.7	2.8	< 0.01
ENSSSCG00000030111			9.1	22.5	2.5	< 0.01
ENSSSCG00000002866	CEBPA	CCAAT/enhancer binding protein alpha	0.9	2.2	2.4	< 0.01
ENSSSCG00000004179	LOC100153984	vascular non-inflammatory molecule 2	0.3	2.1	6.7	< 0.01
ENSSSCG00000021886			1.0	6.4	6.6	< 0.01
ENSSSCG00000003231	NKG7	natural killer cell granule protein 7	1.8	8.7	5.0	< 0.01
ENSSSCG00000010686	BAG3	BCL2 associated athanogene 3	30.6	147.6	4.8	< 0.01
ENSSSCG00000028056	ZFP36	ZFP36 ring finger protein	31.6	140.8	4.5	< 0.01
ENSSSCG00000009012		transmembrane protein 154	2.7	9.9	3.7	< 0.01
ENSSSCG00000026448		tetratricopeptide repeat domain 7B	0.8	2.3	3.0	< 0.01
ENSSSCG00000015541			4.5	10.5	2.4	< 0.01

Ensembl gene ID	Gene name	Gene description	Ctrl	Tx	FC	P
ENSSSCG00000016067	STK17B	serine/threonine kinase 17b	7.8	18.4	2.4	< 0.01
ENSSSCG00000009517	GPR183	G protein-coupled receptor 183	0.9	10.7	11.8	< 0.01
ENSSSCG00000017698	CCL4	C-C motif chemokine 4	4.5	38.2	8.5	< 0.01
ENSSSCG00000014951		pannexin 1	0.6	3.1	5.1	< 0.01
ENSSSCG00000010907	LOC396634	receptor-type tyrosine-protein phosphatase C-like	0.5	2.3	4.9	< 0.01
ENSSSCG00000013553	VAV1	vav 1 guanine nucleotide exchange factor	1.1	5.2	4.9	< 0.01
ENSSSCG00000010806	RGS2-201	Regulator of G-protein signaling 2	47.1	219.5	4.7	< 0.01
ENSSSCG00000001952	NFKBIA-002	NF-kappa-B inhibitor alpha	31.3	120.3	3.9	< 0.01
ENSSSCG00000013237	SPI1		6.4	15.1	2.4	< 0.01
ENSSSCG00000012676	MBNL3	muscleblind like splicing regulator 3	0.7	1.4	2.1	< 0.01
ENSSSCG00000010224		early growth response 2	0.6	4.5	7.6	< 0.01
ENSSSCG00000030165	MAFF	MAF bZIP transcription factor F	31.8	89.0	2.8	< 0.01
ENSSSCG00000029753	CYTIP	cytohesin 1 interacting protein	0.3	3.6	10.6	< 0.01
ENSSSCG00000008937	AMBN	Ameloblastin	0.2	2.0	8.3	< 0.01
ENSSSCG00000026759		heme oxygenase 1	10.7	71.4	6.7	< 0.01
ENSSSCG00000028322	BTG2	BTG family member 2	28.4	164.8	5.8	< 0.01
ENSSSCG00000013735			29.2	161.4	5.5	< 0.01
ENSSSCG00000003155	PPP1R15A	protein phosphatase 1 regulatory subunit 15A	18.6	58.1	3.1	< 0.01
ENSSSCG00000023889	LOC100737500	DNA polymerase eta-like	2.4	6.2	2.5	< 0.01

Ensembl gene ID	Gene name	Gene description	Ctrl	Tx	FC	P

Ensembl gene ID	Gene name	Gene description	Ctrl	Tx	FC	P

Ensembl gene ID	Gene name	Gene description	Ctrl	Tx	FC	P
