# Supplementary material for: Repetitive in vivo manual loading of the spine elicits cellular responses in porcine annuli fibrosi
Source: PLoS One. 2021 Mar 23;16(3):e0248104. doi: 10.1371/journal.pone.0248104 (PMC7987143; doi:10.1371/journal.pone.0248104)
Supplement: Attachment — Submitted filename: reviwer comment.docx [file pone.0248104.s004.docx]

The Manuscript entitled “Repetitive in vivo manual loading of the spine elicits cellular responses in porcine annuli fibrosi” describes the transcriptomic analysis of ventral annuli fibrosi to single dose of spine manual loading. They use a total of 10 replicates to drive the expression of annuli fibrosi cells. The analysis presented in this paper explains, in brief, the functional roles of transcripts after manual loading. In-depth data analysis, such as pathway analysis would have added more value to the paper. Some issues need to be addressed to improve the manuscript overall,

1. The discussion is quite vague and not comprehensive. A major portion of the discussions are on the limitation of the study. A more detailed description of the biological importance of the differentially expressed genes is required.
2. Can the authors explain in brief the functional role of the Annuli fibrosi cells, why these cells were chosen for the study?
3. How many females and males were used? why 3-4 months old were chosen? How relevant it is to use 3-4 months old pig for this study?
4. ketamine, acepromazine, xlyazine, azaperone, and glycopyrrolate? Were all the drugs combined to anesthetize the pigs? What is the dosage used?
5. To identify all majority of DEGs, at least six biological replicates per condition of experiments is recommended. I was wondering why there is a discrepancy in the number of replicates between control and hyperloaded? The hyperloaded pigs are treated differently and have two subgroups. Did the authors make sure that there are no treatment effects within the subgroups of hyper loaded animals? A PCA plot in this case would be useful. I would suggest including PCA plots to see the differences between the groups.
6. No pathological conditions were induced in the pigs before spinal therapy. Apparently healthy pigs were given spinal therapy and gene expressions were compared. The cellular response to spinal therapy in a pathophysiological condition and a healthy individual may vary. The basis of spinal therapy is to relieve pain and molecular changes seen after spinal therapy may shed light on the molecular mechanism of such therapy. Can the author substantiate the reason to perform in healthy animals?
7. A little more careful explanation of the objective, rationale, and the discussion of the study is justified. The overall objective is not clear. The hyperloading was performed to compare the negative effects of spinal manual therapy or to compare the biological changes to spinal manual therapy in diseased state?
8. I would suggest the authors to perform a pathway crosstalk analysis to further characterize the significantly enriched genes-pathways and also to do a co-expression network analysis to see the interaction signature among the significantly DEG.
9. The heat map is of very poor quality. A brief explanation below each figure would be helpful.
