# Supplementary material for: Repetitive in vivo manual loading of the spine elicits cellular responses in porcine annuli fibrosi
Source: PLoS One. 2021 Mar 23;16(3):e0248104. doi: 10.1371/journal.pone.0248104 (PMC7987143; doi:10.1371/journal.pone.0248104)
Supplement: Attachment — Submitted filename: reviewer comment PLoS1_JRM responses v3.docx [file pone.0248104.s005.docx]

Additions are denoted by underlining; ~~deletions denoted by strikeout~~.

*The Manuscript entitled “Repetitive in vivo manual loading of the spine elicits cellular responses in porcine annuli fibrosi” describes the transcriptomic analysis of ventral annuli fibrosi to single dose of spine manual loading. They use a total of 10 replicates to drive the expression of annuli fibrosi cells. The analysis presented in this paper explains, in brief, the functional roles of transcripts after manual loading. In-depth data analysis, such as pathway analysis would have added more value to the paper. Some issues need to be addressed to improve the manuscript overall,*

1. *The discussion is quite vague and not comprehensive. A major portion of the discussions are on the limitation of the study. A more detailed description of the biological importance of the differentially expressed genes is required.*

As with any discovery-based genomic approach, functional biological implications are necessarily second-hand (mRNA cannot resist loads) and hence any interpretation remains speculative. Nonetheless, to address the reviewer’s concerns and to highlight the notable findings of functional significance for the readership, the revised manuscript now includes, along with statements of caution, the use of more specific wording and a fuller discussion of possible biological implications based on the results of our analyses. Specifically, the Discussion has been extensively re-written to more fully compare our findings with those in the literature for a variety of structural and regulatory genes relevant to connective tissues, as well as a synthesis of highly regulated targets with literature on intervertebral disc biology.

2. *Can the authors explain in brief the functional role of the Annuli fibrosi cells, why these cells were chosen for the study?*

The spine moves primarily in flexion-extension in response to dorso-ventral manual therapeutic loading. This loading is resisted primarily by the annulus fibrosus (Kawchuk et al. 2010 [7]), with the ventral annulus forming the apex of spine opposite the applied manual compression load. Hence, the maximal mechanobiological response to dorsal loading is expected to be most evident in cells in the ventral annuli fibrosi.

In the revised manuscript, several specific changes have been made to clarify the choice of these cells (newly added text is underlined):

(a) Introduction: “Previous experimental studies on pig spines, using carefully calibrated robotic movements to reproduce clinical manual therapy, indicate that when manual force is applied to the spine, the intervertebral discs (IVD) receive the largest fraction of the applied load of all spinal tissues [7], and manual loading applied to the dorsal spine is expected to induce maximal loading in the ventral annuli fibrosi.”

(b) Introduction: “Indeed, a remarkable mechanobiology study reports responses by intervertebral disc cells to such IVD loads detectable from even a *single* (1.5 hr) loading event [20], including both upstream (i.e., altered gene expression) as well as downstream effects (i.e., altered tissue concentrations of extracellular matrix proteoglycan and collagen). Notably, these authors report that mRNA changes in annuli fibrosi persist, whereas those in nucleus pulposus were transient [20].”

(c) Introduction: “The present experiments seek to explore the sensitivity of ventral annuli fibrosi cells—the cells responsible for maintaining the structural integrity of the disc—in responding to loads typical of those in clinical practice when applied repeatedly by in vivo spinal manipulation of a porcine model using a discovery-based, broad-spectrum gene expression analysis.”

(d) Methods: “Based on the mid-dorsal application of load, this mid-ventral segment of annulus was chosen for analysis as it was expected to experience the maximum resultant load, thus eliciting the maximal mechanobiological response in the cells of the ventral annuli fibrosi.”

3. *How many females and males were used? why 3-4 months old were chosen? How relevant it is to use 3-4 months old pig for this study?*

Six females and four males were used in these studies (added to Materials section). Sex did not have an appreciable effect on classification of animals into Control and Hyperloaded groups.

For veterinarians and swine producers these studies are practically irrelevant as farm animals are not candidates for veterinary chiropractic therapy, hence, in the context of the present studies the relevance of pigs is primarily as a model of human disease and therapy. As acknowledged in the Discussion, 3-4 month-old pigs while skeletally immature, have attained a suitable size that enables manual loading that simulates clinical therapy in humans (the same cannot be said of smaller animals, nor of much larger animals, including full-grown swine which can reach >300 kg). The choice of animals of this age range was driven primarily by size, both in terms of the anatomical similarity to humans, and by the ability of Dr. Kawchuk to apply manual loads similar to those used routinely on human patients.

The following sentence was modified in the Methods section:

Methods: “Animals ranged in age from 3-4 mos. old and had body mass of 48 kg ± 4 kg (mean ± SD), a size that facilitated the simulation of manual loading in humans.”

Whereas this rationale is based on clinically relevance, this choice is acknowledged as a limitations in the Discussion (page, line):

“Although the use of skeletally immature, quadrupedal pigs as a model of human IVD biology is an obvious limitation, the ~~large~~ size of the pigs in this study enables both precise sampling of IVD tissues and the clinically relevant application of spinal manipulation by a clinician, which makes this model highly advantageous for the purposes of this study.”

4. *ketamine, acepromazine, xylazine, azaperone, and glycopyrrolate? Were all the drugs combined to anesthetize the pigs? What is the dosage used?*

Pigs are notoriously sensitive to anesthetic death, hence, a complex combination of these drugs was used to induce and initially anesthetize the pigs for the duration of these tests. The dosages were devised and applied by the supervising veterinarians based on university standard operating procedures. Starting concentrations were approximately: azaperone (5 mg/kg--sedative); glycopyrrolate (0.01 mg/kg--preanesthetic); maintenance anesthetic mixture: acepromazine (1.1 mg/kg); ketamine (33 mg/kg); xylazine (2 mg/kg). Once induced, animals were maintained throughout the 4 hour application of manual loading on gaseous isofluorane (2%).

5. *To identify all majority of DEGs* (differentially expressed genes)*, at least six biological replicates per condition of experiments is recommended. I was wondering why there is a discrepancy in the number of replicates between control and hyperloaded? The hyperloaded pigs are treated differently and have two subgroups. Did the authors make sure that there are no treatment effects within the subgroups of hyper loaded animals? A PCA plot in this case would be useful. I would suggest including PCA plots to see the differences between the groups.*

Indeed, ideally we would have more replicates to analyse, and the discrepancy in the number of replicates between Hyperloaded and Controls was simply to add additional controls to help assure that a treatment effect could be detected, if possible, with such low numbers.

Given the small numbers, it seemed inappropriate to examine the subgroups which risks both false positives and false negatives given the experimental variance. Rather, it made more sense to us to explore intra-animal comparisons of different levels of loading in the same spine as reported in Table 2.

Nonetheless, on the good suggestion of the reviewers, a principal component analysis was performed which depicts some unexplained variation amongst Non-loaded controls (blue)(possibly related to animal age but not sex), but also depicts a clear and distinct segregation of Non-loaded controls from Hyperloaded (red) samples. Close grouping of Hyperloaded samples suggests there are negligible effects of loading duration and frequency. This figure has been added to the manuscript to make clear this important point.

6. *No pathological conditions were induced in the pigs before spinal therapy. Apparently healthy pigs were given spinal therapy and gene expressions were compared*. The cellular response to spinal therapy in a pathophysiological condition and a healthy individual may vary. *The basis of spinal therapy is to relieve pain and molecular changes seen after spinal therapy may shed light on the molecular mechanism of such therapy. Can the author substantiate the reason to perform in healthy animals?*

As a starting point, we chose to explore the effects of manual loading on a group of healthy controls so as not to confound any treatment effect with pathological changes. Indeed, the present report is designed specifically to serve as the foundation for future work aimed to explore the effects of manual loading on the pathobiology of disc disease. As noted by the reviewers, it is entirely possible and even probable that the response of injured or diseased annuli fibrosi will differ from healthy discs, so this initial study lays the foundation for future studies of diseased intervertebral discs wherein the responses can be compared.

Several changes to help clarify these points are listed under comment #7.

7. A little more careful explanation of the objective, rationale, and the discussion of the study is justified. The overall objective is not clear. The hyperloading was performed to compare the negative effects of spinal manual therapy or to compare the biological changes to spinal manual therapy in diseased state?

In preparing to evaluate the effects of spinal therapy on diseased intervertebral discs, we sought to define very carefully the capacity of healthy disc to respond to a well-defined, “hyperloading” protocol to define if *any* change in gene expression could be elicited in *healthy normal* discs. Such studies are necessary prerequisites for future studies that define: (a) whether cells in healthy annuli fibrosi respond to more modest load histories typical of manual loads used clinically in humans, and (b) whether cells in diseased annuli fibrosi respond in a similar or dissimilar response compared to healthy discs (at various load histories).

In the revised manuscript, these points have been explicitly woven into the rationale and discussion:

(a) Abstract: “The present study defines the cellular response of repeated, low-amplitude loads on normal healthy annuli fibrosi and lays the foundation for future work defining how healthy and diseased intervertebral disc responds to single or low-frequency manual loads typical of those applied clinically.”

(a) Introduction: “In this initial study, a porcine model is used to define any changes in gene expression, as indices of cell responsiveness, in healthy normal annuli fibrosi of positive controls (treated with multiple applications of manual therapy over several hours) and sham-treated (non-loaded) negative controls. These initial proof-of-principle studies can serve as a foundation for future gene expression analyses of normal and diseased annuli fibrosi loaded using clinical doses of manual therapy of the spine.”

(b) Discussion: “Although the use of skeletally immature, quadrupedal pigs as a model of human IVD biology ~~disease~~ is an obvious limitation, the ~~large~~ size of the pigs in this study enables both precise sampling of IVD tissues and the relevant application of spinal manipulation by an experienced clinician, which makes this model highly advantageous for the purposes of this study. .”

(c) Discussion: “The goal here was to define a positive control for applying manual loading to the to the healthy normal IVD, which leaves future studies to determine, whether or not, such molecular changes might also be observed after a typical, clinically applied spinal manipulation in healthy and diseased IVDs.

8. I would suggest the authors to perform a pathway crosstalk analysis to further characterize the significantly enriched genes-pathways and also to do a co-expression network analysis to see the interaction signature among the significantly DEG.

At the reviewers suggestion, the authors undertook further analysis using the Weighted Gene Co-expression Network Analysis (WGCNA) package in R: (<https://horvath.genetics.ucla.edu/html/CoexpressionNetwork/Rpackages/WGCNA/>).

These result of these additional analyses are presented in the revised manuscript. Additional paragraphs are added to the Methods, Results, and Discussion sections to describe and comment these “system-level” findings.

9. The heat map is of very poor quality. A brief explanation below each figure would be helpful.

Whereas the highly detailed heatmap illustrates the subtle differences among animals, the consolidated heatmap shows a striking distinction between Hyperloaded and Non-Loaded Controls. Hence, to emphasize the substantive results to the reader, we have included a new, crisp figure of the consolidated heatmap in the revision. A higher quality detailed heatmap with hundreds of categories is now presented as a Supplemental image.

The figure legends have been updated to include brief explanations.

To minimize confusion, the authors have also elected to delete Figure 2 to avoid duplication and confusion with these data, depending instead on adding statistical analyses to the data presented in Table 2 (comparing two different levels [L_1,2_ vs. L_3,4_] and comparing RT-PCR and RNAseq).
