# Supplementary material for: Repetitive in vivo manual loading of the spine elicits cellular responses in porcine annuli fibrosi
Source: PLoS One. 2021 Mar 23;16(3):e0248104. doi: 10.1371/journal.pone.0248104 (PMC7987143; doi:10.1371/journal.pone.0248104)
Supplement: Attachment — Submitted filename: reviewer comment PLoS1_JRM responses v3.docx [file pone.0248104.s006.docx]

Additions are denoted by underlining; ~~deletions denoted by strikeout~~.

***Reviewer #1***

*The manuscript by Matyas et al., describes the effect of repetitive manual loading applied to the lumbar spine on genes expression in annuli fibrosi cells, to determine whether manual therapy has a positive effect on spine. I think that the manuscript is well written and the idea behind the manuscript is good but I have some major concerns regarding this study.

They used 10 animals (6 Non-loaded and 4 Hyperloaded) but then they decided to use 6 controls and 6 hyperloaded sample for the RNA sequencing analysis as the claim in the methods “The two extra Hyperloaded sample replicates from annulus level L3-4 were powdered, isolated, and purified independently to assess repeatability”. Is not clear if this data were included in the analysis at the end.*

All data were included in these analyses, however these duplicate samples were treated as replicates, i.e., not independent with n=1 for each animal. *Moreover they did not assess the health status of the discs in the animals. Why they did not use also degenerated discs or older animals? I think that if the idea is to understand the effect of manual therapy on spine the assumption should be that individuals that are recommended for therapy have back pain and most probably degenerated discs.*

Only healthy young pigs were used in these studies. Please see the responses to Reviewer #3 for complete responses to these concerns.

*Why they analyze the gene expression changes only in annuli fibrosis and did not analyze also nucleus pulposus cells? It is know that NP cells are important for formation and maintenance of IVD and they are considered signaling center in the IVD (Richardson SM et al., 2017; Hiyama et al., 2013; Winkler T et al., 2014), so would have been a more informative study including also information coming from NP cells. They also did not check the purity of preparation (contamination in AF cells from NP or end plate).*

The reviewer is entirely correct that the nucleus pulposus cells are involved in forming and maintaining the intervertebral disc, and our ongoing and future work on the natural history of disc degeneration includes the nucleus. However, we chose in the present study to focus the analysis on the annulus given its location and predicted loading, and the short-term application of loading. Testing the purity of tissue samples is, as the reviewer knows or suspects, is a rather difficult task for “grind-and-find” experiments such as these. Previous and preliminary experiments using histology and MR imaging support the sampling procedure used here as one that minimizes contamination by endplate cartilage, longitudinal ligaments, and nucleus pulposus. Nevertheless, this is a potential complication of this approach as addressed in the Discussion (page 8).

*Even though the data presented here are indicative of an effect of manual therapy on spine health, additional experiments and data would be needed to support their hypothesis.*

Indeed, these are the first experiments to help define the “physiological window” of annuli fibrosis responsiveness to load. Without these findings as foundation, subsequent analyses of IVDs receiving clinical loads would lack an anchor.

***Reviewer #2***

*The manuscript entitled “Repetitive in vivo manual loading of the spine elicits cellular responses in porcine annuli fibrosis” by Matyas et al., analyzed the response of annuli fibrosis for in vivo loading using a transcriptomic approach. The study is interesting and uncovered the differential expression of genes involved in the metabolic process. Although there are certain limitations (due to model and variable response of IVD to loading and corresponding cellular response) in the study which the authors fairly pointed in the discussion studies of similar kind in future will lay the foundation to understand the molecular and biochemical response to physical forces in treating back pain. As the authors rightly concluded there is a need for additional studies in the field to confirm the observed transcriptome regulation to interpret the results clinically.

Overall the manuscript is well written, easy to understand, and adequately detailed for the methods followed. I recommend accepting this manuscript with a minor revision.

Minor suggestions.*

*1. At the outset how do the authors rule out the observed changes in mRNA regulations are not due to stress applied during the hyper loading procedure?*

There are two types of stress in these studies: one is the stress of anesthesia and positioning; another is the physical stress of hyperloading itself. As all animals receiving the same anesthesia and duration, whatever stresses are induced by these procedures are controlled but not evaluated. It is noteworthy that physical restraint and euthanasia without anesthesia may also induce (an unknown) stress. The experimental controls in these experiments were non-loading “shams”, i.e., the laying on of hands without delivery of manual loading. *2. Since the current study can stand as a reference to future studies on IVD loading Is it possible for the authors to group or cluster the differentially regulated genes and show how the regulated genes interact (using tools like DAVID for interaction analysis) with genes differentially regulated in IVD degenerative disease data sets? This will support to claim that the genes regulated after the short term repetitive IVD loading procedure could compensate for the clinical need.*

As described in the comment #8 by Reviewer#3, we performed weighted gene co-expression network analysis (WGCNA) that unbiasedly clusters the differentially expressed genes. Currently there has not been a comprehensive analysis of differentially expressed genes in diseased discs (only reports on selected targets) nor any kind of co-expression network analysis.

*3. The authors can move Figure 1A to supplement and keep Figure 1B as main Figure 1.*

Done. *4. The authors can represent the qPCR data as a bar graph in Figure 2 along with the RNA seq fold change. Label the genes in the RNA seq Fold change panel. This data can also be combined as panel B and C in Figure 1.*

We have deleted Figure 2 as it is confusing and duplicates the data in Table 2.

*5. I believe Fig 3 is a representation of Table 4, If not what is the difference in the analysis?*

Table 4 is a list of gene ontologies that are *statistically over- and under-represented* in differentially expressed genes *compared to baseline*; Fig 3 enumerates and compares the *total number* of genes both up- and down-regulated in each of 31 standard GO categories. While similar, these representations are not congruent. *6. From the GO analysis, I see the upregulation of genes involved in immune response which is not very well discussed in the manuscript. The upregulated immune genes or pathways could be key for the therapy? please discuss.*

The WGCNA analysis presented in Table 5 reveals that the blue module is essentially a collection of genes that support the core functions of the innate immune system. This reinforces the highly ranked DAVID clusters listed in Table 3. This immune system stimulus is explicitly discussed in the Discussion (last paragraph of page 11). *7. Also, the pictorial representation of gene ontology in Fig 3 should be verified for the functional terms on Y-axis.*

Whereas Fig 3 enumerates the numbers of genes changing (up and down) with each biological, molecular, or cellular process, the functional significance of differentially expressed genes in various pathways is resolved by the WGCNA analyses.  *8. The authors cited Table S1 and S2 for differentially regulated genes, but I could not find the supplement file in the submission.*

These have been included in the resubmission and are available in the public repository:

*Open Science Framework (OSF) data repository (Center for Open Science):*

*Identifier: DOI 10.17605/OSF.IO/75NUG

9. Please provide the GEO accession for the data set submission*

Accession number: GSE166656

*10. Dataset submitted to OSF needs to be curated carefully for missing gene name and details*

The data in OSF while having a number of omissions is the most up-to-date annotation of the porcine genome.

***Reviewer#3***

*The Manuscript entitled “Repetitive in vivo manual loading of the spine elicits cellular responses in porcine annuli fibrosi” describes the transcriptomic analysis of ventral annuli fibrosi to single dose of spine manual loading. They use a total of 10 replicates to drive the expression of annuli fibrosi cells. The analysis presented in this paper explains, in brief, the functional roles of transcripts after manual loading. In-depth data analysis, such as pathway analysis would have added more value to the paper. Some issues need to be addressed to improve the manuscript overall,*

1. *The discussion is quite vague and not comprehensive. A major portion of the discussions are on the limitation of the study. A more detailed description of the biological importance of the differentially expressed genes is required.*

As with any discovery-based genomic approach, functional biological implications are necessarily second-hand (mRNA cannot resist loads) and hence any interpretation remains speculative. Nonetheless, to address the reviewer’s concerns and to highlight the notable findings of functional significance for the readership, the revised manuscript now includes, along with statements of caution, the use of more specific wording and a fuller discussion of possible biological implications based on the results of our analyses. Specifically, the Discussion has been extensively re-written to more fully compare our findings with those in the literature for a variety of structural and regulatory genes relevant to connective tissues, as well as a synthesis of highly regulated targets with literature on intervertebral disc biology.

2. *Can the authors explain in brief the functional role of the Annuli fibrosi cells, why these cells were chosen for the study?*

The spine moves primarily in flexion-extension in response to dorso-ventral manual therapeutic loading. This loading is resisted primarily by the annulus fibrosus (Kawchuk et al. 2010 [7]), with the ventral annulus forming the apex of spine opposite the applied manual compression load. Hence, the maximal mechanobiological response to dorsal loading is expected to be most evident in cells in the ventral annuli fibrosi.

In the revised manuscript, several specific changes have been made to clarify the choice of these cells (newly added text is underlined):

(a) Introduction: “Previous experimental studies on pig spines, using carefully calibrated robotic movements to reproduce clinical manual therapy, indicate that when manual force is applied to the spine, the intervertebral discs (IVD) receive the largest fraction of the applied load of all spinal tissues [7], and manual loading applied to the dorsal spine is expected to induce maximal loading in the ventral annuli fibrosi.”

(b) Introduction: “Indeed, a remarkable mechanobiology study reports responses by intervertebral disc cells to such IVD loads detectable from even a *single* (1.5 hr) loading event [20], including both upstream (i.e., altered gene expression) as well as downstream effects (i.e., altered tissue concentrations of extracellular matrix proteoglycan and collagen). Notably, these authors report that mRNA changes in annuli fibrosi persist, whereas those in nucleus pulposus were transient [20].”

(c) Introduction: “The present experiments seek to explore the sensitivity of ventral annuli fibrosi cells—the cells responsible for maintaining the structural integrity of the disc—in responding to loads typical of those in clinical practice when applied repeatedly by in vivo spinal manipulation of a porcine model using a discovery-based, broad-spectrum gene expression analysis.”

(d) Methods: “Based on the mid-dorsal application of load, this mid-ventral segment of annulus was chosen for analysis as it was expected to experience the maximum resultant load, thus eliciting the maximal mechanobiological response in the cells of the ventral annuli fibrosi.”

3. *How many females and males were used? why 3-4 months old were chosen? How relevant it is to use 3-4 months old pig for this study?*

Six females and four males were used in these studies (added to Materials section). Sex did not have an appreciable effect on classification of animals into Control and Hyperloaded groups.

For veterinarians and swine producers these studies are practically irrelevant as farm animals are not candidates for veterinary chiropractic therapy, hence, in the context of the present studies the relevance of pigs is primarily as a model of human disease and therapy. As acknowledged in the Discussion, 3-4 month-old pigs while skeletally immature, have attained a suitable size that enables manual loading that simulates clinical therapy in humans (the same cannot be said of smaller animals, nor of much larger animals, including full-grown swine which can reach >300 kg). The choice of animals of this age range was driven primarily by size, both in terms of the anatomical similarity to humans, and by the ability of Dr. Kawchuk to apply manual loads similar to those used routinely on human patients.

The following sentence was modified in the Methods section:

Methods: “Animals ranged in age from 3-4 mos. old and had body mass of 48 kg ± 4 kg (mean ± SD), a size that facilitated the simulation of manual loading in humans.”

Whereas this rationale is based on clinically relevance, this choice is acknowledged as a limitations in the Discussion (page, line):

“Although the use of skeletally immature, quadrupedal pigs as a model of human IVD biology is an obvious limitation, the ~~large~~ size of the pigs in this study enables both precise sampling of IVD tissues and the clinically relevant application of spinal manipulation by a clinician, which makes this model highly advantageous for the purposes of this study.”

4. *ketamine, acepromazine, xylazine, azaperone, and glycopyrrolate? Were all the drugs combined to anesthetize the pigs? What is the dosage used?*

Pigs are notoriously sensitive to anesthetic death, hence, a complex combination of these drugs was used to induce and initially anesthetize the pigs for the duration of these tests. The dosages were devised and applied by the supervising veterinarians based on university standard operating procedures. Starting concentrations were approximately: azaperone (5 mg/kg--sedative); glycopyrrolate (0.01 mg/kg--preanesthetic); maintenance anesthetic mixture: acepromazine (1.1 mg/kg); ketamine (33 mg/kg); xylazine (2 mg/kg). Once induced, animals were maintained throughout the 4 hour application of manual loading on gaseous isofluorane (2%).

5. *To identify all majority of DEGs* (differentially expressed genes)*, at least six biological replicates per condition of experiments is recommended. I was wondering why there is a discrepancy in the number of replicates between control and hyperloaded? The hyperloaded pigs are treated differently and have two subgroups. Did the authors make sure that there are no treatment effects within the subgroups of hyper loaded animals? A PCA plot in this case would be useful. I would suggest including PCA plots to see the differences between the groups.*

Indeed, ideally we would have more replicates to analyse, and the discrepancy in the number of replicates between Hyperloaded and Controls was simply to add additional controls to help assure that a treatment effect could be detected, if possible, with such low numbers.

Given the small numbers, it seemed inappropriate to examine the subgroups which risks both false positives and false negatives given the experimental variance. Rather, it made more sense to us to explore intra-animal comparisons of different levels of loading in the same spine as reported in Table 2.

Nonetheless, on the good suggestion of the reviewers, a principal component analysis was performed which depicts some unexplained variation amongst Non-loaded controls (blue)(possibly related to animal age but not sex), but also depicts a clear and distinct segregation of Non-loaded controls from Hyperloaded (red) samples. Close grouping of Hyperloaded samples suggests there are negligible effects of loading duration and frequency. This figure has been added to the manuscript to make clear this important point.

6. *No pathological conditions were induced in the pigs before spinal therapy. Apparently healthy pigs were given spinal therapy and gene expressions were compared*. The cellular response to spinal therapy in a pathophysiological condition and a healthy individual may vary. *The basis of spinal therapy is to relieve pain and molecular changes seen after spinal therapy may shed light on the molecular mechanism of such therapy. Can the author substantiate the reason to perform in healthy animals?*

As a starting point, we chose to explore the effects of manual loading on a group of healthy controls so as not to confound any treatment effect with pathological changes. Indeed, the present report is designed specifically to serve as the foundation for future work aimed to explore the effects of manual loading on the pathobiology of disc disease. As noted by the reviewers, it is entirely possible and even probable that the response of injured or diseased annuli fibrosi will differ from healthy discs, so this initial study lays the foundation for future studies of diseased intervertebral discs wherein the responses can be compared.

Several changes to help clarify these points are listed under comment #7.

7. A little more careful explanation of the objective, rationale, and the discussion of the study is justified. The overall objective is not clear. The hyperloading was performed to compare the negative effects of spinal manual therapy or to compare the biological changes to spinal manual therapy in diseased state?

In preparing to evaluate the effects of spinal therapy on diseased intervertebral discs, we sought to define very carefully the capacity of healthy disc to respond to a well-defined, “hyperloading” protocol to define if *any* change in gene expression could be elicited in *healthy normal* discs. Such studies are necessary prerequisites for future studies that define: (a) whether cells in healthy annuli fibrosi respond to more modest load histories typical of manual loads used clinically in humans, and (b) whether cells in diseased annuli fibrosi respond in a similar or dissimilar response compared to healthy discs (at various load histories).

In the revised manuscript, these points have been explicitly woven into the rationale and discussion:

(a) Abstract: “The present study defines the cellular response of repeated, low-amplitude loads on normal healthy annuli fibrosi and lays the foundation for future work defining how healthy and diseased intervertebral disc responds to single or low-frequency manual loads typical of those applied clinically.”

(a) Introduction: “In this initial study, a porcine model is used to define any changes in gene expression, as indices of cell responsiveness, in healthy normal annuli fibrosi of positive controls (treated with multiple applications of manual therapy over several hours) and sham-treated (non-loaded) negative controls. These initial proof-of-principle studies can serve as a foundation for future gene expression analyses of normal and diseased annuli fibrosi loaded using clinical doses of manual therapy of the spine.”

(b) Discussion: “Although the use of skeletally immature, quadrupedal pigs as a model of human IVD biology ~~disease~~ is an obvious limitation, the ~~large~~ size of the pigs in this study enables both precise sampling of IVD tissues and the relevant application of spinal manipulation by an experienced clinician, which makes this model highly advantageous for the purposes of this study. .”

(c) Discussion: “The goal here was to define a positive control for applying manual loading to the to the healthy normal IVD, which leaves future studies to determine, whether or not, such molecular changes might also be observed after a typical, clinically applied spinal manipulation in healthy and diseased IVDs.

8. I would suggest the authors to perform a pathway crosstalk analysis to further characterize the significantly enriched genes-pathways and also to do a co-expression network analysis to see the interaction signature among the significantly DEG.

At the reviewers suggestion, the authors undertook further analysis using the Weighted Gene Co-expression Network Analysis (WGCNA) package in R: (<https://horvath.genetics.ucla.edu/html/CoexpressionNetwork/Rpackages/WGCNA/>).

These result of these additional analyses are presented in the revised manuscript. Additional paragraphs are added to the Methods, Results, and Discussion sections to describe and comment these “system-level” findings.

9. The heat map is of very poor quality. A brief explanation below each figure would be helpful.

Whereas the highly detailed heatmap illustrates the subtle differences among animals, the consolidated heatmap shows a striking distinction between Hyperloaded and Non-Loaded Controls. Hence, to emphasize the substantive results to the reader, we have included a new, crisp figure of the consolidated heatmap in the revision. A higher quality detailed heatmap with hundreds of categories is now presented as a Supplemental image.

The figure legends have been updated to include brief explanations.

To minimize confusion, the authors have also elected to delete Figure 2 to avoid duplication and confusion with these data, depending instead on adding statistical analyses to the data presented in Table 2 (comparing two different levels [L_1,2_ vs. L_3,4_] and comparing RT-PCR and RNAseq).
